# A gram-positive enhancer matrix particles vaccine displaying swine influenza virus hemagglutinin protects mice against lethal H1N1 viral challenge

**DOI:** 10.3389/fimmu.2024.1432989

**Published:** 2025-01-06

**Authors:** Yufei Zhang, Pei Zhang, Xiaoyue Du, Xiaona Shi, Jinling Wang, Shuying Liu

**Affiliations:** ^1^ College of Veterinary Medicine, Inner Mongolia Agricultural University, Hohhot, China; ^2^ Inner Mongolia Key Laboratory of Basic Veterinary Science, Inner Mongolia Agricultural University, Hohhot, China; ^3^ Key Laboratory of Clinical Diagnosis and Treatment Technology in Animal Disease, Ministry of Agriculture, Hohhot, China

**Keywords:** influenza, swine influenza, vaccine, intranasal vaccine, gram-positive enhancer matrix (GEM) particles

## Abstract

**Introduction:**

Animal influenza viruses pose a danger to the general public. Eurasian avian-like H1N1 (EA H1N1) viruses have recently infected humans in several different countries and are often found in pigs in China, indicating that they have the potential to cause a pandemic. Therefore, there is an urgent need to develop a potent vaccine against EA H1N1.

**Methods:**

In this study, we report the effective intramuscular (i.m.) and/or intranasal (i.n.) vaccination of mice with a subunit influenza vaccine utilizing safe adjuvant gram-positive enhancer matrix (GEM) particles derived from the food-grade bacterium *Lactococcus lactis*. The hemagglutinin (HA)-protein anchor (PA) subunit vaccine can be simply mixed with GEM particles to produce vaccines.

**Results:**

After two booster injections, the i.m.+i.n. administered GEM subunit vaccine achieved hemagglutination inhibition titers in the serum that were equivalent to those observed using the conventional i.m. method. The mucosal and Th1-biased immune responses generated by the i.m. administered subunit vaccine alone were inferior to those induced by the i.n. and i.m.+i.n. administered subunit vaccines. Vaccinated mice were challenged with live viruses (G4 EA H1N1 and A/PR/8/34) to determine whether the adjuvant combination protected against the virus after vaccination with the influenza subunit vaccine. Compared to mice inoculated with HA alone, mice immunized with i.m.+i.n. or i.n. HA-PA-GEM displayed undetectable viral titers in the lungs, at 5 d after challenge.

**Discussion:**

Overall, this study not only offers other potential platforms for the generation of swine influenza vaccines, but also a theoretical foundation for vaccine vector platforms that can be utilized for future research on other infections.

## 1 Introduction

Due to their vulnerability to avian, swine, and human influenza A viruses (IAVs), pigs are regarded as “mixing vessels” for the creation of influenza viruses with the potential to cause a pandemic (1–4). Moreover, these reassortants may evolve mutations to adapt to humans, resulting in a human influenza pandemic (5). With the emergence of pdm/09H1N1, the global health threat posed by novel swine influenza viruses (SIVs) has been clearly illustrated (5, 6). There are several genotypes of Eurasian avian-like H1N1 (EA H1N1) swine IAVs in pigs in China, and recent findings suggest that the potentially pandemic genotype 4 (G4) reassortment has been predominant among swine populations in China since 2016 (4, 7, 8).

Despite its low mortality in swine herds, swine influenza is an economically significant respiratory infectious disease that causes high morbidity in pigs worldwide (7). Prevention of H1N1 SIVs will contribute to improving public health by reducing the risk of transmission from pigs to humans. Effective immunization is currently the most affordable public health measure for controlling SIV infections. Recently, various influenza vaccines have been developed for pigs, including inactivated whole-virus, live attenuated, subunit, and vectored vaccines (9, 10). Inactivated whole-virus vaccines are currently the most used and commercially accessible option for preventing SIV infections (11). Since influenza virus outbreaks are caused by the antigenic structure of the virus, hemagglutinin (HA) is a crucial component in the design of a vaccine against them (12). HA antigenic matching between the vaccine strain and prevalent strain is essential for the inactivated influenza vaccine to effectively protect against epidemic strains.

Vaccines using inactivated viruses and/or subunit vaccines frequently contain adjuvants to boost the strength and caliber of immune responses, while also accelerating the onset and lengthening the duration of protection. Numerous adjuvants can boost immune responses and encourage defense against infections with influenza virus strains that are similar to one another, in both humans and animals (9). The food-grade bacterium *Lactococcus lactis* (*L. lactis*) is heated and acidified to remove its DNA and most of its proteins, resulting in the formation of peptidoglycan spheres known as gram-positive enhancer matrix (GEM) nanoparticles (13, 14). This novel adjuvant technology of GEM particles can significantly increase the immunogenicity of vaccines (15–20). Toll-like receptor 2 recognizes GEM particles, which activate innate immunity and improve the capacity of the natural immune system to kill harmful microorganisms (13, 20). It has been found that the GEM-protein anchor (PA) surface display system, which uses a PA made from the *L. lactis* peptidoglycan hydrolase, AcmA, may efficiently elicit systemic immune responses, in addition to offering other advantages, such as quick and simple antigen purification (15–20). Recent studies of GEM particles have demonstrated their safety in the treatment of a number of pathogens, including shigellosis, human papillomavirus, porcine circovirus type 2, hepatitis E virus and foot-and-mouth disease virus (15–20). GEM particles are safe and effective adjuvants for intranasal (i.n.) and intramuscular (i.m.) injections during influenza subunit vaccination (20).

In this study, we examined how well BALB/c mice responded to i.m. and i.n. administration of an influenza subunit vaccine used in combination with GEM particles as an adjuvant. For this, we tested whether (a) the GEM particles had adjuvant/immunostimulatory activity for intranasal immunization; (b) the adjuvants enhanced mucosal and systemic immune responses to influenza; and (c) the mice were protected against a fatal EA H1N1 swine IAV challenge through the passive administration of influenza formulations that were or were not adjuvanted.

## 2 Materials and methods

### 2.1 Viruses, cells, and bacterial strains

The G4 EA H1N1 swine isolate [A/swine/Jiangsu/65/2015(H1N1)] and influenza virus A/PR/8/34 (HIN1) were kindly provided by Meilin Jin (21). Allantoic inoculation of the seed virus was performed to cultivate the virus in embryonated chicken eggs. The virus was purified and inactivated as previously described, to obtain whole-inactivated virus (WIV) vaccines (22, 23). In brief, the G4 EA H1N1 swine isolate culture supernatant was treated with 0.1 M β-propiolactone (BPL) (P5648, MilliporeSigma, USA) for overnight viral inactivation at 4°C. BPL was then hydrolyzed at 37°C for 90 minutes. Inactivated viruses were concentrated by ultracentrifugation at 50,000 g for 2 h and purified using a 20-60% sucrose density gradient. Total protein of the purified viruses was quantified with a bicinchoninic acid (BCA) protein assay kit (Sangon Biotech, Shanghai, China). Madin-Darby canine kidney (MDCK) and RAW 264.7 (mouse macrophage) cells were obtained from the American Type Culture Collection (ATCC). The cells were cultured in Dulbecco’s Modified Eagle’s Medium (Gibco, Shanghai, China), at 37°C, in an incubator containing 5% CO_2_. Adherent Spodoptera frugiperda (Sf9) cells (Life Technologies, USA) were cultured at 27°C and maintained in SFM 900 II medium (Life Technologies, Beijing, China) supplemented with 10% heat-inactivated fetal bovine serum and 1% penicillin-streptomycin. *L. lactis* MG1363 was purchased from the China Committee for Culture Collection of Microorganisms (Beijing, China). Standing cultures of the cells were maintained at 30°C in M17 medium containing 1% glucose.

### 2.2 GEM preparation

The *L. lactis* strain MG1363 was cultured overnight and harvested. The cells were then washed with sterile distilled water, following which hydrochloric acid (HCl; 0.1 M) was added to them, and they were placed in a hot water bath for 30 min, at 99°C. The bacteria were killed by treatment with acid and heat, to generate the GEM particles. After three washes with PBS, the GEM particles were diluted to one unit (1 U=2.5×10^9^ particles/mL) and stored at −70°C.

### 2.3 Transmission and scanning electron microscopy

For transmission electron microscopy, the *L. lactis* strain MG1363 and GEM particles were centrifuged to obtain precipitates, which were reconstituted in sterile filtered water following fixation with 2% glutaraldehyde (no suspension), overnight at 4°C. A simple carbon grid was used to hold the *L. lactis* MG1363 and GEM formulations. After placing on the grid, the samples were washed with water and stained twice with 5 µL of 2 wt% uranyl acetate. Images of the samples were captured with an UltraScan 4000SP CCD camera (Gatan).

Particles of *L. lactis* strains MG1363 and GEM were deposited on a metal disc using double-sided adhesive carbon tape and coated with approximately 10 nm of gold using a Balzer’s 120B sputtering apparatus (Balzer UNION, Liechtenstein). Following that, the *L. lactis* strain MG1363 and GEM preparations were imaged using a JEOL JSM 6301-F microscope (JEOL Ltd., Tokyo, Japan), at magnifications of 500× and 5000×.

### 2.4 Particle size analysis

A Litesizer™ 500 system (Anto Paar, Austria) was used to analyze the particle size of the *L. lactis* strain MG1363 and GEM particles (at a dilution factor of 1:100), with distilled water used as the diluent.

### 2.5 Nitrite assay

The nitrite assay has been described in detail elsewhere (24). RAW 264.7 cells were stimulated with 0.1, 0.5, or 1 U doses of GEM particles, and the culture supernatants were collected 48 h after incubation, to measure the nitric oxide (NO) levels. While the cells in the negative control group received only cell culture medium, those in the positive control groups were stimulated with Pam3CK4 or lipopolysaccharide (LPS), at concentrations of 0.1, 1, or 10 µg/mL. Griess assay was used to measure NO production, according to the manufacturer’s protocol (Promega, Madison, WI, USA).

### 2.6 Quantitative real-time PCR

Total RNA extraction from the samples was carried out using TRIzol^®^ (Invitrogen, Beijing, China), as per the manufacturer’s instructions. Conventional and quantitative real-time PCR (qRT-PCR) assays were conducted on the extracted RNA following cDNA synthesis. SYBR^®^ Premix Ex Taq™ (Tli RNase H Plus; Takara, Dalian, China) was used for qRT-PCR, to examine the expression of nitric oxide synthase (iNOSl), interleukin (IL)-1, interferon gamma (IFN-γ), tumor necrosis factor alpha (TNF-α), and IL-6 on an ABI 7500 system (Applied Biosystems, USA). Relative gene expression was analyzed based on the threshold cycle (2^–ΔΔCT^) method, with GAPDH used as an internal control. Three independent experiments were conducted. The qRT-PCR primers used are listed in **Supplementary Figure 1** (21, 25).

### 2.7 Immunohistology

All mouse heads were fixed in 10% neutral-buffered formalin for 48 h, after being skinned. Six pieces were cut from the tip of the nose to the foramen occipitale magnum using a diamond saw. The sections were gently decalcified for 7 d, at room temperature, in an RDF Mild Decalcifier (CellPath Ltd., The United Kingdom). The tissues (lungs and thymus) were fixed in 10% neutral-buffered formalin for 72 h, before being transferred to 70% ethanol for paraffin embedding. The implanted tissues were routinely divided into sections that were 3~4 µm thick, stained with Masson’s trichrome as well as hematoxylin and eosin, and then examined by a veterinary pathologist. As previously described (4, 25, 26), immunohistochemistry was carried out using the horseradish peroxidase (HRP) method. Sections of 5 μm were cut, deparaffinized in xylene, rehydrated in graded alcohol, and microwaved in sodium citrate buffer (pH 6.0) for antigen retrieval. Endogenous peroxidase activity was blocked with 3% H_2_O_2_ for 15 min, and the lung tissue sections were then blocked with 5% BSA for 30 min, both at room temperature. The samples were incubated with Rat anti-mouse cluster of differentiation (CD)45R (clone B220; BD Biosciences; B cells), rabbit anti-CD3 [ab16669; Abcam (Shanghai, China); T cells], and rabbit anti-Iba-1 (ab178847; Abcam; macrophages and dendritic cells) for 14 h at 4°C. The slides were incubated with a secondary antibody using the ImmPRESS™ HRP Universal Antibody Polymer Detection Kit (Vector Laboratories, MP-7500) for 1 hour at room temperature. After 25 seconds of DAB staining and 30 seconds of counterstaining with hematoxylin in 1% ammonia solution, the slides were mounted with VectaMount medium, examined, and photographed using an Olympus BX41 microscope. Images were acquired using cellSens software (Olympus). 5 random sections were chosen for the intensity quantification. The marker staining intensities were evaluated by relative quantification using digital image analysis platform DefiniensTissueStudio (Definiens AG).

### 2.8 Construction and expression of recombinant baculoviruses

**Figure 1:**
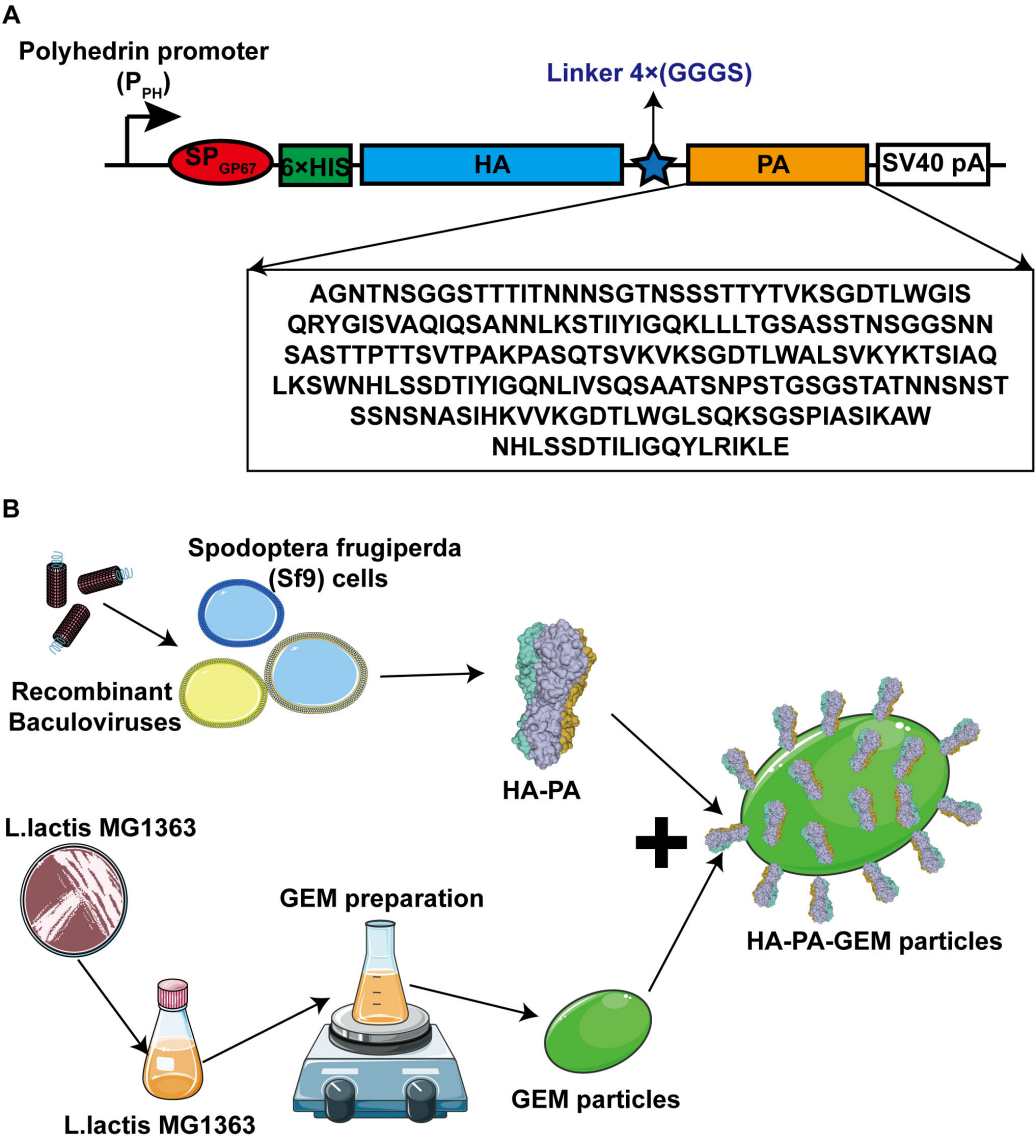
Schematic diagrams of antigen design and HA-PA protein binding to GEM particles. **(A)** The secreted signal peptide gp67 was added to the N-terminal of HA in both the constructs. The PA sequence was added after the HA sequence, and a 6×His tag was added to the C-terminal of gp67. **(B)** Combination diagram of HA-PA protein binding to GEM particles. HA, hemagglutinin; GEM, gram-positive enhancer matrix; PA, protein anchor.

The HA sequence (GenBank: OL468248.1) of the G4 EA H1N1 swine isolate and PA sequence from the *L. lactis* MG1363 strain were retrieved and synthesized by Sangon Biotech (Shanghai, China). A 6×His-tag and flexible linker sequence were added to the N- and C-termini of the HA gene, respectively, and then cloned into the pFastBac1 vector, immediately downstream of a cassette encoding an envelope glycoprotein signal peptide (gp64) from the nucleopolyhedrovirus *Autographa californica* (**Figure 1A**). A full-length HA protein without the signal peptide, transmembrane, or internal motif was expressed in this study, corresponding to amino acids 20–530 (GenBank: OL468248.1).

PFastBac1-HA-PA was transformed into *Escherichia coli* DH10Bac competent cells to generate recombinant bacmids. The recombinant baculovirus was created by transiently transfecting the recombinant bacmid into Sf9 insect cells with Cellfectin^®^ II (Life Technologies), as directed by the manufacturer. First-generation (P1) recombinant baculovirus-containing supernatants were collected 72 h after transfection and passed through three generations of Sf9 cells to produce the fourth-generation (P4) recombinant baculoviruses. In the culture supernatant, HA-PA was expressed as a soluble secreted protein. To express recombinant proteins, 2×10^6^ cells/mL of Sf9 cells were infected with amplified high-titer recombinant HA-PA baculoviral stocks at 5% (a multiplicity of index of ~3). Expression was carried out at 27°C for 72 h, on an orbital shaker rotating at 120 rpm. Similarly, HA expression was done in Sf9 cells using the pFastBac baculovirus system. The procedure was identical to the above. The only difference between HA and HA-PA is the missing PA tag. The protein was purified from the supernatant while the cell pellet was discarded.

### 2.9 Purification of recombinant proteins

To optimize the expression of recombinant HA-PA or HA protein in scaled-up 1L cultures, Sf9 cells were cultured in shaking flasks with orbital rotation at 125 rpm at 27°C and subsequently infected with P3 baculoviral stock. After 72 hours, the recombinant HA-PA or HA protein was purified from the supernatant, with the cell pellet being discarded. The purification process was conducted using Ni-NTA-affinity chromatography (BIOFOUNT, Beijing, China) in accordance with the manufacturer’s instructions. The supernatant was subjected to purification using a Ni-NTA column (10 mL, 1.6 × 5 cm) at a flow rate of 1 mL/min. The column was sequentially washed with washing buffer 1 (PBS, pH 7.4), washing buffer 2 (25 mmol/L imidazole in PBS, pH 7.4), and washing buffer 3 (70 mmol/L imidazole in PBS, pH 7.4). Subsequently, the recombinant HA-PA or HA was eluted using a stepwise imidazole gradient ranging from 200 to 400 mM in PBS (pH 7.4). The concentration of the purified HA-PA or HA was quantified using the bicinchoninic acid (BCA) assay.

### 2.10 Binding of the fusion protein to GEM particles

The HA-PA-GEM particles were assembled in a single step at room temperature, as shown in **Figure 1B**. Three days after injecting the recombinant baculovirus into Sf9 cells at a multiplicity of index of 3, the cells were harvested by means of centrifugation (1600 × *g* for 30 min), and the supernatant was collected. The purification process was conducted using Ni-NTA-affinity chromatography (BIOFOUNT, Beijing, China) in accordance with the manufacturer’s instructions. Subsequently, 5 µg/µL HA-PA fusion protein was mixed with 1 U GEM particles (2.5×10^9^) at room temperature, for 1 h, in a rotational shaker. To acquire the fusion-GEM complexes, the precipitate was centrifuged at 6000 × *g* for 10 min, washed five times with PBS, and then resuspended in PBS. Samples were then analyzed by Western blot.

### 2.11 Western blot and immunofluorescence assay

To study antibody binding, the proteins were resolved on a 10% w/v polyacrylamide gel and then transferred to a polyvinylidene difluoride membrane. The blots were blocked with 5% skim milk powder in PBS before being incubated with 0.5 µg/mL of an anti-6×His-tag monoclonal antibody diluted in 1% skim milk powder in PBS, overnight at 4°C. The blots were then incubated with HRP-conjugated rabbit anti-mouse immunoglobulin G (IgG) from Abcam (diluted 1:10000 with 1% skimmed milk powder in PBS) for 1 h, at room temperature. Chemiluminescence was detected using the Clarity Western ECL Substrate Kit (Bio-Rad, UK). To further evaluate the antigenicity of HA-PA, positive sera from A/swine/Jiangsu/65/2015 (H1N1) were employed as the primary antibody. The sera were obtained 20 days following infection of pigs with A/swine/Jiangsu/65/2015 (H1N1). The secondary antibody employed was goat anti-pig IgG H&L (Abcam, ab6910, Shanghai, China).

For the immunofluorescence assay (IFA), the centrifuged HA-PA-GEM complex precipitate was suspended and blocked with 5% non-fat milk in Eppendorf tubes. Next, the cells were stained with an anti-6×His-tag monoclonal antibody (1:200) as the primary antibody and FITC-conjugated anti-mouse IgG (1:400) as the secondary antibody. A fluorescence microscope was used to view the dyed complexes on slides (Olympus Corp., Tokyo, Japan).

### 2.12 Hemagglutination assay

Hemagglutination assays were performed as described previously (16). The HA-PA-GEM complex were reconstituted in PBS to a concentration of 25 µg/mL, and 50 µL of this preparation was added to 96-well V bottom plates already containing 50 µL of PBS. Following a two-fold serial dilution of the entire mixture, 50 µL of a 1.5% adult chicken erythrocyte suspension were added to each well, and the plates were then stored at room temperature for 2 h. The maximum dilution at which red blood cell agglutination was observed was used to express the hemagglutination titers, which were read after 2 h.

### 2.13 Animal study design

#### 2.13.1 Vaccine immunizing evaluation

The Committee for Animal Experiments of the Inner Mongolia Agricultural University in China examined and authorized the animal research, in compliance with the provisions of the Chinese Animal Protection Act. BALB/c mice (6–8-weeks old) were purchased from Beijing Vital River Laboratory Animal Technology Co. Ltd. The female mice were divided into nine groups (i.n. PBS group, i.m. PBS group, i.n. GEM group, i.m. GEM group, i.m. HA subunit vaccine group, i.m. WIV, i.m. HA-PA-GEM group, i.n. HA-PA-GEM group, i.m.+i.n. HA-PA-GEM group) of eight each. These experimental animals are mainly used to study the immune response to vaccines. In order to immunize mice with an i.m. vaccine, a dose of the vaccine contained 1.5 μg of whole-inactivated virus antigen in 25 μL of phosphate-buffered saline (PBS) containing 25 μL of adjuvant AddaVax (vac-adx-10, InvivoGen) (per mouse in 50 μL). HA subunit vaccine group mice were injected with HA protein (0.2 μg/μL) was mixed with an equal volume of incomplete Freund’s adjuvant (Sigma-Aldrich, Shanghai, Chain) (per mouse in 50 μL). Immediately before vaccination, the GEM particles and HA-PA were mixed. Under inhalation anesthesia (isoflurane/O_2_), the test group received 25 µL of GEM-adjuvanted vaccination, through the i.n. or/and i.m. routes [12 uL of the 1U per mL of GEMs combined with 5 µg HA-PA]. The animals were administered the vaccine on three occasions, at days 0, 14, and 28, via the intramuscular route and subsequently humanely euthanized on day 40 of the trial.

On day 40, the mice were euthanized, after which their orbital plexus was punctured to collect blood for antibody measurements. Bronchoalveolar lavage fluid was collected to assess the secretory IgA (SIgA) antibodies. Briefly, the lungs from sacrificed mice were flushed three times with 1 mL PBS, then centrifuged at 3,500 g for 10 minutes. Roche’s “complete” protease inhibitor was used in 1 mL of PBS, as per the manufacturer’s instructions, for the bronchoalveolar lavage fluid. All lavage samples were stored at –20°C.

#### 2.13.2 Viral challenge

In the present study, two animal studies were performed to investigate protection against heterologous challenge by H1N1 viruses with different antigens (**Supplementary Figure 1**). In study 1, 80 BALB/c mice aged 6-8 weeks were divided into 10 groups (**Supplementary Figure 2**). The intramuscular vaccination groups included mock vaccine control group (PBS), HA subunit vaccine group (HA), WIV group, GEM group and HA-PA-GEM group. These mice were nasally inoculated with the G4 EA H1N1 swine isolate (1×10^5^ TCID_50_/0.1 mL) via 2.5% avertin (0.02 mL/g body weight) 1 week after the third immunization, and negative controls were treated with 0.9% saline. The intranasal vaccination groups included blank control (PBS), HA subunit vaccine (HA) group, GEM group, HA-PA-GEM group, i.m.+i.n. HA-PA-GEM group. Similarly, these mice were nasally inoculated with the G4 EA H1N1 swine isolate (1×10^5^ TCID_50_/0.1 mL) via 2.5% avertin (0.02 mL/g body weight) 1 week after the third immunization, and the negative control group was treated with 0.9% saline.

In study 2, 48 BALB/c mice aged 6-8 weeks were divided into 6 groups (**Supplementary Table 2**). The intramuscular vaccination groups included blank control (PBS), WIV group and HA-PA-GEM group. These mice were nasally inoculated with influenza virus A/PR/8/34 (HIN1) (1×10^5^ TCID_50_/0.1 mL) via 2.5% avertin (0.02 mL/g body weight) 1 week after the third immunization, and the negative control group was treated with 0.9% saline. The intranasal vaccination groups included blank control (PBS), HA-PA-GEM group, i.m.+i.n. HA-PA-GEM group. Similarly, these mice were nasally inoculated with influenza virus A/PR/8/34 (HIN1) (1×10^5^ TCID_50_/0.1 mL) via 2.5% avertin (0.02 mL/g body weight) 1 week after the third immunization and the negative control group was treated with 0.9% saline.

### 2.14 Enzyme-linked immunosorbent assay for detection of HA-specific antibodies

Serum, and bronchoalveolar lavage fluid samples were collected on 12 day after 3rd immunization, and ELISA was performed to detect HA-specific antibodies in them, as described previously (21). Antigen-specific serum IgG and SIgA antibodies were also detected using ELISA. Whole-inactivated H1N1 virus (100 μL/well) was diluted to a concentration of 10 μg/mL in carbonate-bicarbonate buffer (pH 9.6), and then used to coat 96-well plates, overnight at 4°C. Following that, the wells were blocked with 250 μL of 2% bovine serum albumin in PBS, at 37°C, for 3 h. We diluted the serum samples 100-fold and lavage samples 20-fold. The diluted samples were added to the wells, maintained to 37°C for 2 h, and then rinsed three times with PBS containing Tween-20. The plates were blotted with 100 μL each of HRP-conjugated goat anti-mouse IgG and IgA alpha chain antibodies from Abcam, for 1 h, at room temperature. After this incubation, the wells were washed three times with a 100 μL wash solution. After 15 min of dark incubation at 37°C with a 3,3,5,5′-tetramethylbenzidine substrate solution in sterile water, the bound antibodies were detected. After addition of 100 μL of stop solution to halt the enzyme process, the absorbance of each microwell at the wavelength of 450 nm was measured using a spectrophotometer. Two independent tests were performed.

### 2.15 Hemagglutination inhibition and microneutralization assays

We carried out an HAI test to assess the titers of HA antigen-specific antibodies in the sera or bronchoalveolar lavage fluid of unvaccinated and immunized mice. To inactivate nonspecific inhibitors, the serum samples were pre-treated with a receptor-destroying enzyme (RDE) obtained from Denka Seiken Company, Chuo, Tokyo, before testing. Specifically, three volumes of RDE were combined with one volume of serum and incubated overnight at 37°C. Subsequently, the RDE was inactivated by incubating the serum-RDE mixture at 56°C for approximately 45 minutes. Before being incubated in 96-well microtiter plates with 4 HA units for 30 min at 37°C, the serum or bronchoalveolar lavage fluid samples were serially diluted twice with PBS. Thereafter, an equal volume of fresh, 1% (v/v), chicken red blood cells were added, and the mixture was incubated at 37°C for 30 min. For descriptive and analytic statistics, the assay limit of detection was set at 1:10 for HAI titers. The HAI titer was measured from the highest dilution showing non-agglutinated RBCs. Each plate included positive and negative serum controls. Initially, mice had no antibodies (HAI titer <1:10) against the four vaccine viruses. Positive control pig sera (G4 EA H1N1 swine isolate) and negative controls (antigen-alone wells and PBS with RBCs) were used in the assay.

The viral neutralization experiment was performed as mentioned previously (21). Serum neutralizing antibody titers were measured by seeding 1.5×10^4^ MDCK cells per well into 96-well culture plates, and growing them at 37°C in a 5% CO_2_-containing incubator, to form a monolayer. Serial two-fold dilutions of blood samples were prepared in 96-well cell culture plates containing Dulbecco’s Modified Eagle’s Medium with 0.3% bovine serum albumin, 100 U/mL penicillin, and 100 μg/mL streptomycin (starting dilution of 1:10). Thereafter, an identical volume (50 μL) of diluted virus with 100 TCID_50_ was fixed to the diluted serums, and the mixture was incubated for 1 h, at 37°C. We added 100 μL of serum and virus mixture to 96-well plates containing 90% confluent monolayers of MDCK cells, and then incubated the plates at 37°C for 48 hours. After 48 h, the cell supernatant was collected and an hemagglutination test was carried out.

### 2.16 Splenocyte proliferation and cytokine assay

Splenocytes were harvested from the spleens of three mice (The samples were collected on 12 day after 3rd immunization) and their proliferation was measured (21). For this, splenocytes (1.0 × 10^6^) were seeded into each well of a 96-well microwell plate, with recombinant H1N1 HA antigen (the G4 EA H1N1 swine isolate, GenBank: OL468248.1) (10 ng/mL) as the specific antigen for the vaccine groups and concanavalin A (10 ng/mL) as the positive control, or without stimulation. They were then stimulated for 48 h at 37°C in a humidified environment containing 5% CO_2_. Each mouse sample was examined in triplicate. The MTT assay was used to measure cell viability (21).

Splenocyte stimulation and cytokine analysis were performed as previously described (21). We evaluated the concentrations of Th1 (IFN-γ and IL-12) and Th2 (IL-4) cytokines in the supernatants of splenocytes from control and test animals, in accordance with the manufacturer’s instructions, using commercially available cytokine-specific ELISA kits (R&D Systems, Shanghai, China).

### 2.17 IFN-γ and IL-4 enzyme-linked immunosorbent spot assays

Splenocytes were isolated from the vaccinated mice after the third vaccination. In a 96-well ELISpot plate, 1×10^6^ splenocytes were grown with pure H1N1-HA antigen (the G4 EA H1N1 swine isolate, GenBank: OL468248.1) (20 μg/mL). The cells were grown for 24 h in preparation for the ELISpot assay and then detected using a commercial kit (MABTECH, Nacka, Sweden), in accordance with the manufacturer’s instructions. The spot-forming cells were counted using an automated ELISpot reader (ELISPOT reader iSpot, AID, Germany).

### 2.18 Viral challenge, clinical observation, and histopathological examination

We anaesthetized mice with 2.5% avertin (0.02 mL/g body weight) and then i.n. inoculated them with G4 EA H1N1 swine isolate or A/PR/8/34 (HIN1) (1×10^5^ TCID_50_/0.1 mL). All mice were monitored for general physical activity and pathophysiological measures, to determine the protective effects of the HA-PA-GEM particles (body weight, fur ruffling, and conjunctivitis). Clinical scoring was conducted by an individual who was blinded to both the study design and the identity of the animals. The scoring was based on the following scale: 0 = no visible signs of disease; 1 = slight ruffling of fur; 2 = ruffled fur with reduced mobility (2 points for 10-15% weightloss); 3 = ruffled fur, reduced mobility, and rapid breathing (3 points for 15-20% weightloss); 4 = ruffled fur, minimal mobility, huddled appearance, and rapid and/or labored breathing (4 points for 20-30% weightloss); 5 = euthanasia (A weight loss of greater than 30% was deemed to be a reason for euthanasia.).

The mice were observed daily for signs of distress by monitoring their general appearance, respiratory distress, weight loss and animal survival. Mice that lost more than 30% of their initial body weight were humanely euthanized by carbon dioxide inhalation and cervical dislocation. The sole criterion employed to ascertain whether mice should be euthanized was their body weight. One half of the lung was fixed for histopathological analysis. One half of the lung of virus were titered using median tissue culture infectious dose 50 (TCID 50) assay. For histopathological analysis, lungs were collected on the seventh day post-infection (n = 2), then inflated and fixed with 10% neutral buffered formalin (NBF). For TCID 50 analysis, the lungs were weighed before homogenization in Hanks balanced salt solution (HBSS) and centrifugation at 500 g for 5 minutes. With MDCK cells, TCID 50 tests were conducted to determine virus titers.

Mice from each group had their lungs preserved in 10% neutral-buffered formalin, embedded in paraffin according to accepted practices, sectioned to a thickness of 4 mm, and stained with hematoxylin and eosin. The slides were examined under a light microscope (EX200, Nikon) to detect histological lung lesions. The participants in the evaluation were all postgraduate students in veterinary pathology, and none of them had any prior knowledge of the experiment. The histological assessment criteria for lung damage are categorized into three distinct groups, yielding a cumulative score of up to 10 points per specimen. The evaluation of pulmonary edema involves an examination of both the location and severity of edema within the lung tissue, with a scoring range from 0 (indicating no edema) to 3 (indicating diffuse alveolar space edema affecting multiple lung lobes). Specifically, the scoring is delineated as follows: a score of 1 denotes alveolar wall edema confined to a single lobe, a score of 1.5 indicates alveolar wall edema present in more than one lobe, a score of 2 represents diffuse edema within a single lobe, and a score of 3 corresponds to diffuse edema across multiple lobes. Alveolar infiltration is assessed to determine the extent of infiltration within the alveolar septa and spaces. The evaluation utilizes a scoring system ranging from 0 to 3, where 0 indicates no infiltration and 3 denotes diffuse alveolar space infiltration affecting more than one lobe. The specific scores are defined as follows: a score of 1 corresponds to peribronchiolar and/or perivascular infiltration; a score of 1.5 indicates similar infiltration with localized involvement of the alveolar walls; a score of 2 represents significant tissue consolidation within a single lobe; and a score of 3 signifies extensive infiltration across multiple lobes. Pulmonary vasculitis quantifies the extent of inflammation within vascular structures, utilizing a scoring system that ranges from 0 (indicating no inflammation) to 4 (denoting extensive infiltration across multiple lobes). The criteria for these scores are as follows: a score of 1 corresponds to the presence of perivascular edema and/or infiltration; a score of 2 indicates mild infiltration of the vessel wall without endothelial involvement; a score of 3 signifies intensive infiltration of the vessel wall and/or endothelium confined to a single lobe; and a score of 4 reflects similar intensive infiltration affecting multiple lobes.

### 2.19 Statistical analysis

The data were collected from at least three different experiments and displayed as mean ± standard error of mean. The assays were conducted in triplicate, with at least three independent biological replicates. The reactions were performed in triplicate on each of two biological replicates. Differences were examined using Prism (GraphPad version 5.0). One-way analysis of variance with Bonferroni’s *post-hoc* multiple comparison test was used to determine the statistical significance of differences in cytokine, IgG and SIgA levels between the experimental groups at different time-points. The survival percentages were examined using the Kaplan-Meier technique. A *p*-value of ≤0.05 was considered statistically significant.

## 3 Results

### 3.1 Induction of NO production and gene expression in macrophages by GEM particles

Hot TCA treatment of *L. lactis* MG1363 resulted in the generation of non-living particles (GEM particles), most likely by influencing the protein and DNA content of the GEM particles. The GEM particles had the same size and shape as live *L. lactis* MG1363 (**Supplementary Figures 2A–D**). Hot TCA treatment causes unraveling of the peptidoglycan, which is widely found on the cell wall of *L. lactis* MG1363 and preserves its structural integrity (13–20).

**Figure 2:**
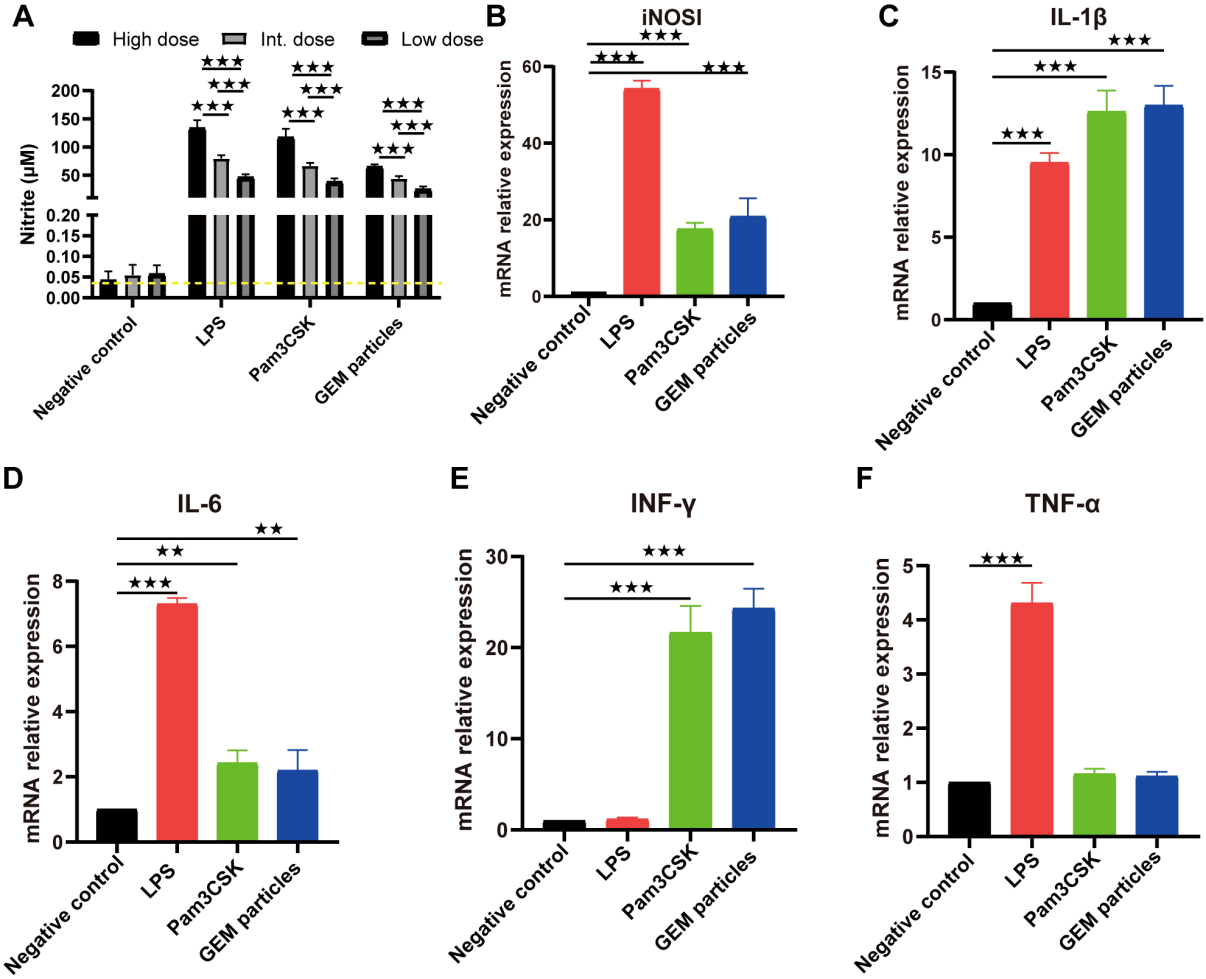
Nitrite production and mRNA expression in RAW 264.7 cells stimulated with GEM particles. **(A)** Nitrite levels in the supernatants were measured using the Griess assay, after 48 h of stimulation. RT-qPCR was performed for iNOSl **(B)** and the cytokines IL-1β **(C)**, IL-6 **(D)**, IFN-γ **(E)**, and TNF-α **(F)**. The gene expression was quantitated relative to that of the housekeeping gene GAPDH. The analysis was performed with a one-way ANOVA. Differences were considered significant at **p<0.01, ***p<0.001, as compared to the respective control cells. The experiment was performed in triplicate. iNOSl, nitric oxide synthase; IL, interleukin; IFN-γ, interferon gamma; TNF-α, tumor necrosis factor alpha; GEM, gram-positive enhancer matrix.

The objective of this study was to investigate the impact of GEM particles on macrophages. To this end, RAW 264.7 cells were stimulated with doses of GEM particles at 0.1, 0.5, or 1 U, and the culture supernatants were collected 48 hours after incubation to measure the nitric oxide (NO) levels. RAW 264.7 cells were treated with three different concentrations of LPS or Pam3CK4 (as positive controls) for 48 h. NO production was measured as a marker of macrophage activation. GEM-stimulated macrophages produced considerably higher nitrite concentrations than unstimulated controls (*p*<0.001) (**Figure 2A**).

We also examined the iNOSl, IL-1β, IL-6, IFN-γ, and TNF-α mRNA levels in the GEMs(1U)-, LPS(10 µg/mL)-, and Pam3CK4(10 µg/mL)-stimulated RAW 264.7 macrophage cells. At 12 h post-treatment, iNOSl expression in cells treated with GEMs, LPS, and Pam3CK4 was upregulated by 20.8- (*p*<0.001), 54.2- (*p*<0.001), and 17.6-fold (*p*<0.001), respectively (**Figure 2B**); IL-1β expression was upregulated by 13- (*p*<0.001), 9.5- (*p*<0.001), and 12.6-fold (*p*<0.001), respectively (**Figure 2C**); IL-6 expression was upregulated by 2.2- (*p*<0.01), 7.3- (*p*<0.001), and 2.4-fold (*p*<0.01), respectively (**Figure 2D**); IFN-γ expression was upregulated by 24.3- (*p*<0.001), 1.2- (*p*>0.05) and 21.7-fold (*p*<0.001), respectively (**Figure 2E**); and TNF-α expression was upregulated by 1.1- (*p*>0.05), 4.3- (*p*<0.001) and 1.2-fold (*p*>0.05), respectively (**Figure 2F**). These results indicated that GEM particles activated macrophages to induce inflammatory cytokines.

### 3.2 GEM particles have inherent adjuvanticity

**Figure 3:**
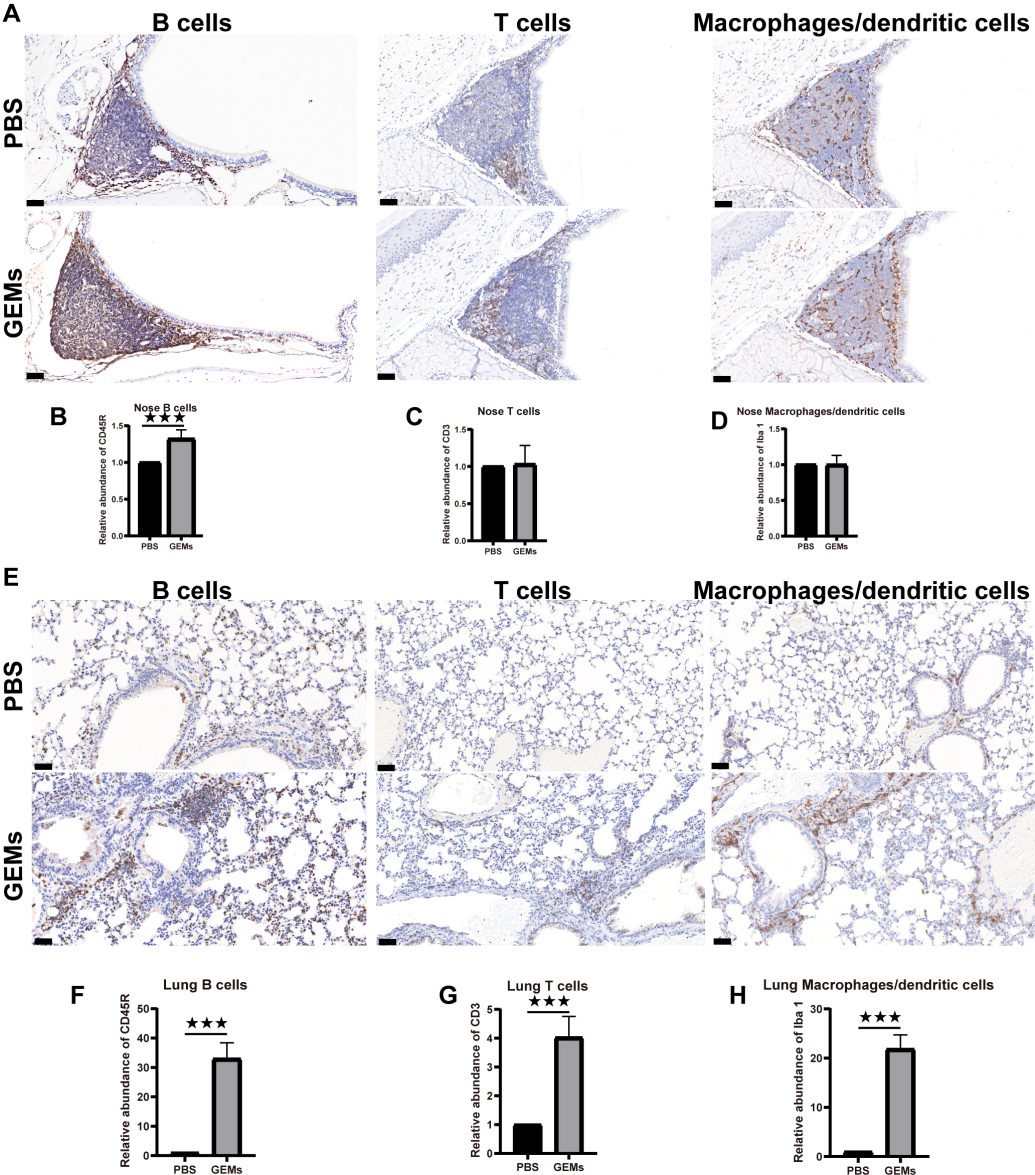
Intrinsic adjuvanticity of the GEMs. **(A)** The mice were intranasally administered PBS alone or GEMs in PBS. Five days later, the mice heads were processed for immunohistology, to visualize immune cell activation and formation of organized lymphoid tissue containing CD45R+ B cells (B220) **(B)**, CD3+ T cells (CD3), (C) and macrophages/dendritic cells (Iba-1) (D) in the nasal-associated lymphoid tissue. **(E)** The lungs were processed for immunohistology, to visualize immune cell activation and formation of organized lymphoid tissue containing CD45R+ B cells (B220) **(F)**, CD3+ T cells (CD3) **(G)**, and macrophages/dendritic cells (Iba-1) **(H)** in the bronchus-associated lymphoid tissue. Statistical analyses were performed using t tests. Differences were considered significant at ***p<0.001, as compared to the respective control groups. PBS, phosphate-buffered saline; CD, cluster of differentiation; GEM, gram-positive enhancer matrix.

Adjuvants are frequently added to inactivated, split virus vaccines, and nanoparticulate subunit vaccines, to improve their immunogenicity and reduce the number of doses and amount of antigen (or pathogen component) required to elicit a protective immune response, particularly in immunocompromised individuals (27–29). i.n. administration of GEM in PBS was used to test the adjuvant effects of the GEMs in this study. Under inhalation anesthesia (isoflurane/O_2_), the test group received of GEM, through the i.n. routes [2.5×10^9^ GEM particles (1 U)]. The control group was substituted with PBS. The head and thoracic organs were removed *en bloc* after 5 d and histologically and immunohistologically investigated (**Figure 3**; **Supplementary Figure 3**) for the presence of organized lymphoid structures and follicles that signify an active immune response. Both the nasal cavity (nasal-associated lymphoid tissue) in **Figures 3A–D** and lungs (bronchus-associated lymphoid tissue, BALT) in **Figures 3E–H** feature large structured lymphoid follicles, including dendritic cells, T cells, and a considerable number of B cells. It was demonstrated that the administration of GEM via the intranasal route in mice resulted in an increase in the number of B cells within the nasal passages and lungs, as well as T cells and dendritic cells in lungs. The number of nasal T cells and dendritic cells remained unaltered. These immunological changes were not observed in mice treated with PBS alone (**Figure 3**). We also evaluated the cytotoxic effects of various doses of GEM administered i.n. (**Supplementary Figure 4**). The findings suggest that the quantity of GEM particles employed should not exceed 5 U (equivalent to 2.5 × 10^9^ GEM particles, or 1 U) per mouse (20 g) to guarantee the well-being of the mice. In conclusion, GEM particles have inherent adjuvanticity and native GEM particles can be i.n. administered with no negative health consequences and no tissue pathology visible in the treated animals postmortem, from a bio-safety perspective.

### 3.3 Expression of H1N1 HA-PA fusion protein and identification of HA-PA-GEM particles

**Figure 4:**
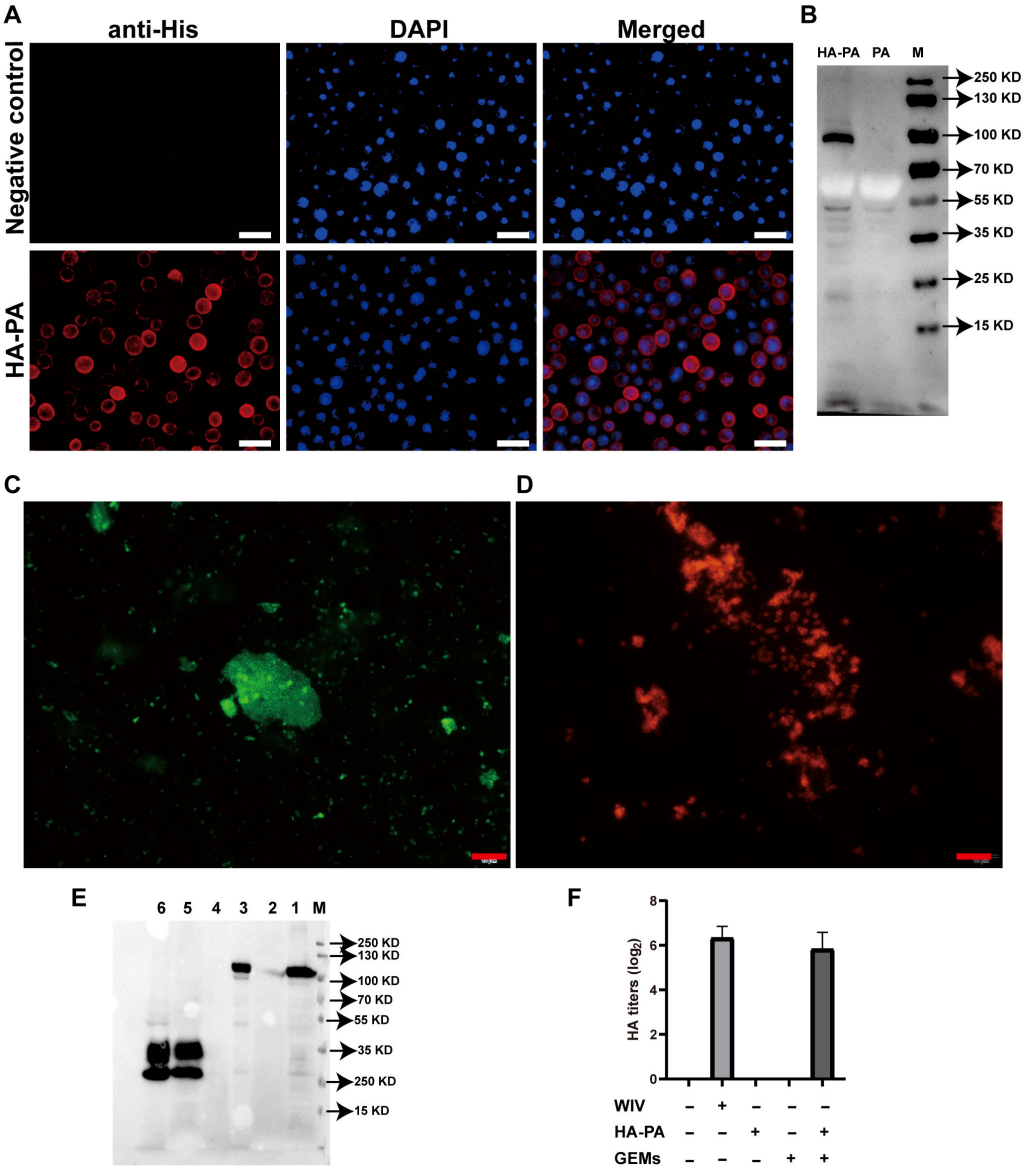
Investigation of fusion-GEM-complex binding. **(A)** IFA of HA-PA expression in baculovirus-infected Sf9 insect cells. **(B)** Western blot analysis of antibody specificity. Lane M: molecular weight marker; Lane PA: recombinant baculovirus (PA)-infected Sf9 cell lysate; Lane HA-PA: recombinant baculovirus (HA-PA)-infected Sf9 cell lysate. **(C)** Fusion proteins on the surface of GEM particles were detected using IFA. Immunofluorescence was detected as green fluorescence using anti-6×His-tag monoclonal antibody and FITC-conjugated goat anti-mouse antibody, while **(D)** red fluorescence was detected in samples seropositive from swine H1N1, upon staining with DyLight 594-conjugated goat anti-swine IgG. **(E)** The maximum binding capacity of the fusion protein displayed on GEM particles was determined using western blot. Lane M: protein marker. Lane 1: recombinant baculovirus (HA-PA)-infected Sf9 cell lysate. Lane 2: 1 U GEM particles. Lane 3: 1 U HA-PA-GEM particles. Lane 4: Sf9 cell lysate. Lane 5: recombinant baculovirus (PA)-infected Sf9 cell lysate. Lane 6: 1 U PA-GEM particles. **(F)** The HA activity of WIV, HA-PA, GEM particles or HA-PA-GEM particles. IFA, immunofluorescence analysis; HA, hemagglutinin; PA, protein anchor; GEM, gram-positive enhancer matrix.

Transfection of Sf9 cells with the recombinant bacmid pFBac-H1N1-HA-PA led to effective recovery of the recombinant baculovirus, Bac-H1N1-HA-PA. Compared to the control cells, Sf9 cells expressing the HA-PA fusion protein showed strong red fluorescence (**Figure 4A**). Moreover, a 100 kDa band matching the HA-PA fusion protein was detected upon western blot analysis of the cell lysate from Sf9 cells infected with the recombinant baculovirus, thus demonstrating its expression (**Figure 4B**). This result suggested that the HA-PA protein reacted well with seropositive swine H1N1 samples, with good antigenicity.

HA-PA-GEMs were subjected to both immunofluorescence (**Figures 4C, D**) and western blot (**Figure 4E**) analyses, to confirm whether the HA-PA fusion protein was capable of binding to GEM particles via PA. Immunofluorescence analysis results showed that, compared to GEM particles alone (**Supplementary Figure 5A**), the combination of GEM particles and HA-PA fusion protein emitted strong green fluorescence (**Figure 4C**) upon staining with anti-6×His-tag monoclonal antibody. Likewise, compared to GEM particles alone (**Supplementary Figure 5B**), the combination of GEM particles and HA-PA fusion protein emitted strong red fluorescence (**Figure 4D**) in samples seropositive for swine H1N1. In addition, western blot analysis confirmed that the GEM particles alone contained no detectable HA-PA fusion protein, as expected. HA-PA-GEMs confirmed the presence of the H1N1-HA-PA fusion protein (**Figure 4E**). The HA-PA fusion protein was linked to GEM particles according to the aforementioned data. Furthermore, an HA assay was performed to determine whether the receptor-binding activity of HA was still present. The inclusion of GEM particles resulted in an increase in the hemagglutination titers, thereby demonstrating that GEM particles had a positive impact on the biological activity of HA (**Figure 4F**).

### 3.4 Serum influenza-specific IgG levels after immunization with HA-PA-GEM particles

**Figure 5:**
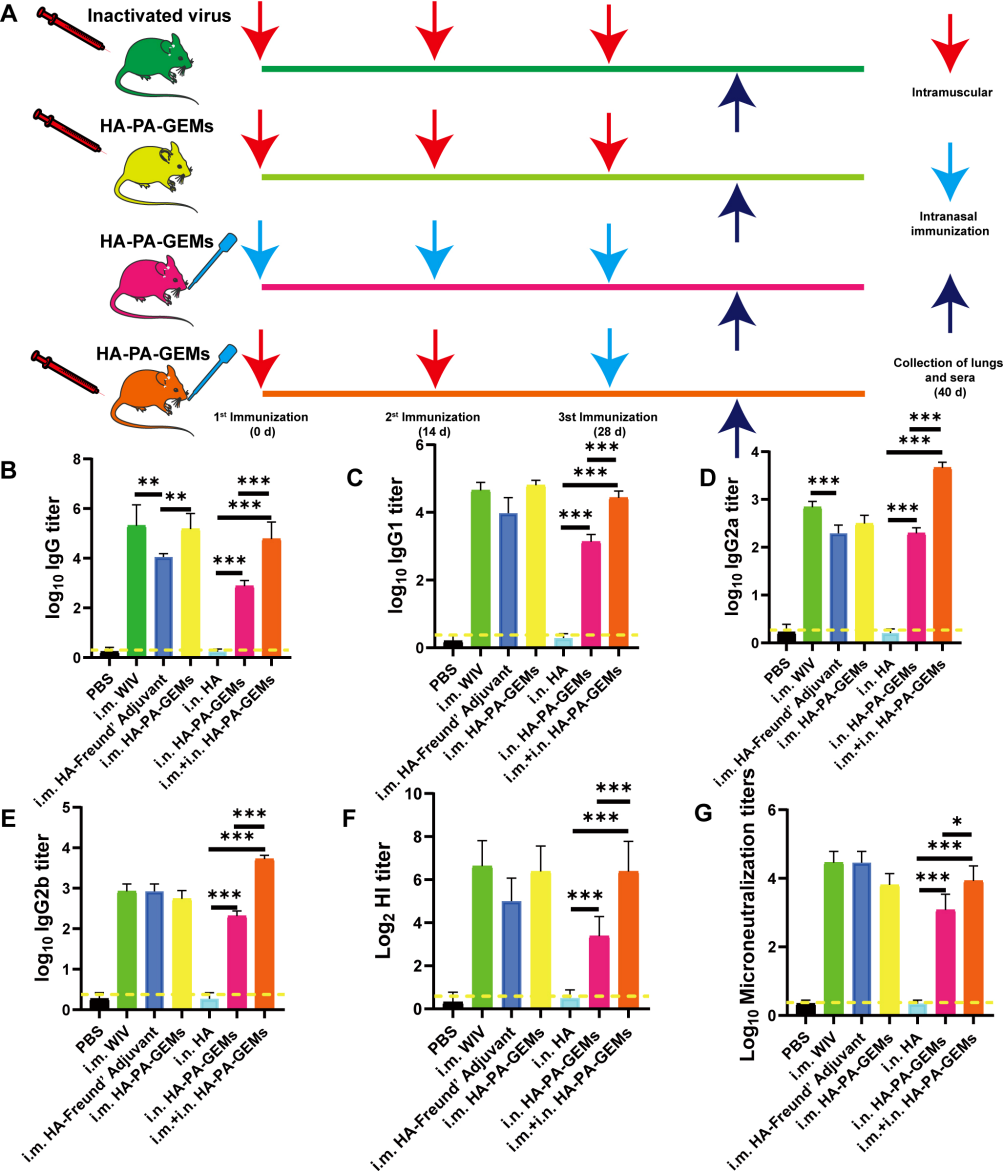
Serum antibody levels induced by the GEM-adjuvanted H1N1 vaccine. **(A)** Experimental protocol for i.m. and i.n. immunization of mice with HA-PA-GEMs and inactivated virus. HA-specific **(B)** IgG, **(C)** IgG1, **(D)** IgG2a, **(E)** IgG2b levels in the serum were assessed using indirect ELISA. **(F)** HI and **(G)** micro-neutralization titers in the serum. The dotted yellow line shows the limit of detection. Dotted line indicate a 1:1.5 HAI titer. The analysis was performed with a one-way ANOVA. Differences were considered significant at *p<0.05, **p<0.01, ***p<0.001. GEM, gram-positive enhancer matrix; i.m., intramuscular; i.n., intranasal; HA, hemagglutinin; PA, protein anchor; ELISA, enzyme-linked immunosorbent assay; HI, hemagglutination inhibition.

i.m. or i.n. immunization with HA-PA-GEM particles was performed on eight groups of mice, with control animals receiving PBS alone. The animals in each group received a primary vaccination, two follow-up immunizations at 2 and 4 weeks later, and weekly serum collection until one week after the third repeat immunization (**Figure 5A**). After immunization, the mice exhibited no abnormalities or negative effects. BALB/c mice immunized with HA-PA-GEM particles were found to have HA protein-specific IgG specific to the G4 EA H1N1 swine isolate (**Figure 5B**), while those immunized with PBS did not.

The response was further characterized by determining IgG isotypes. At week 6, we measured the quality of the humoral response using IgG2a, IgG2b, and IgG1 subtype-specific H1N1 ELISAs (**Figures 5C–E**). According to earlier research (4–6), a strong IgG1 response was induced by subunit vaccine i.m. immunization, but low IgG2a and IgG2b responses occurred, indicating a skewed Th2-type response. After i.n.+i.m. immunization with HA-PA-GEM, IgG2a and IgG2b responses were significantly higher (*p*<0.001) compared to i.m. immunization with HA-PA-GEM, while IgG1 responses were significantly lower (*p*<0.001) compared to i.m. immunization with HA-PA-GEM. HA-PA-GEM induced a different nature of immunity biased to Th1- and Th2-type, respectively, as judged by the ratio of H1N1-specific IgG isotypes (IgG2a/IgG1 and IgG2b/IgG1) (**Supplementary Figure 6**). Based on these results, we concluded that HA-PA-GEM vaccination induced antibody responses with a Th1 phenotype, which were markedly different from those induced by traditional i.m. vaccination.

Serum HI titers were measured to further evaluate systemic immune responses. After the third booster shot, the HI titers for each mouse were measured. I.n. HA-PA-GEM formulations showed a trend toward lesser HI titers than similar the WIV vaccine after the third immunization (**Figure 5F**). Even after the third booster shot, vaccination with the WIV vaccine alone produced same HI titers. Microneutralization titers were consistent with those of HI (**Figure 5G**).

### 3.5 Mucosal influenza-specific SIgA antibody levels after immunization with HA-PA-GEM particles

**Figure 6:**
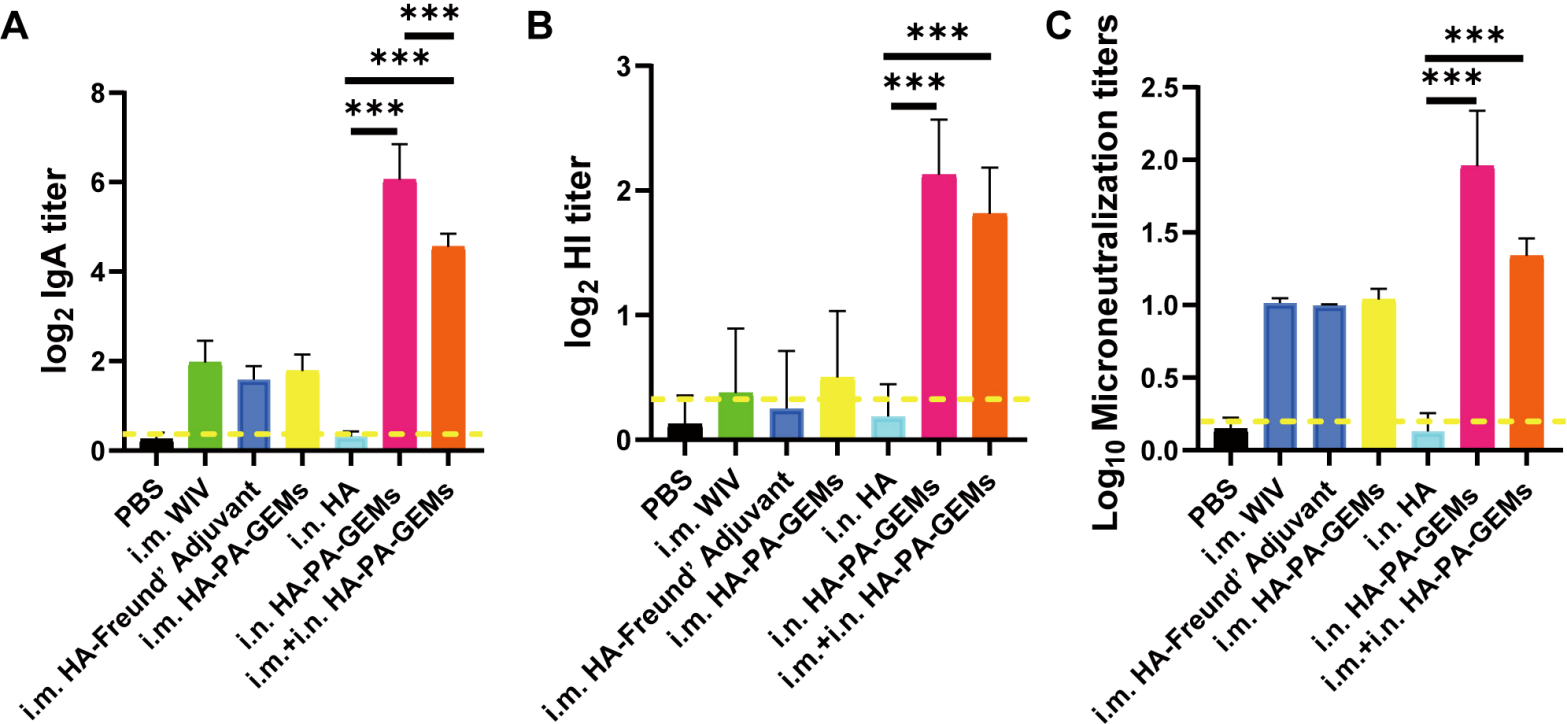
IgA, HAI and microneutralization titers induced by GEM-adjuvanted H1N1 vaccines. Bronchoalveolar lavage fluid was collected 12 day after the third immunization, for detection of HI and virus neutralization antibody titers. **(A)** HA-specific IgA and **(B)** HI antibody titers were determined upon challenge with swIAV H1N1 virus, and the results were calculated as log2. **(C)** Virus neutralization antibody titers were determined against 100 TCID50 swIAV H1N1 virus, and the results were calculated as log10. The dotted yellow line shows the limit of detection. Dotted line indicate a 1:1.5 HAI titer. The analysis was performed with a one-way ANOVA with Bonferroni’s post-hoc multiple comparison test. Differences were considered significant at ***p<0.001. GEM, gram-positive enhancer matrix; HA, hemagglutinin; HI, hemagglutination inhibition; IgA, immunoglobulin A; PA, protein anchor; WIV, whole-inactivated virus; TCID_50_, median tissue culture infectious dose.

IAV infection occurs mainly through mucosal tissues, whereas SIgA from the mucosa can block IAV infection. Therefore, we tested the ability of the vaccine to induce mucosal immunity (**Figure 6A**). In most mice, i.m. immunization resulted in SIgA levels below the limit of detection in the lung lavage. Furthermore, the lung lavage produced low SIgA titers following immunization with the subunit vaccine alone. In contrast, i.n. vaccination with HA-PA-GEM induced high SIgA levels in the lungs of all the mice (*p*<0.001). Furthermore, we measured HI (**Figure 6B**) and microneutralization titers (**Figure 6C**) for these samples, which maintained a tendency consistent with the aforementioned relationship. Finally, we concluded that i.n. HA-PA-GEM immunization induced a strong mucosal airway immune response.

### 3.6 Splenocyte proliferation after *ex vivo* stimulation and antigen-specific T-cell immune responses

**Figure 7:**
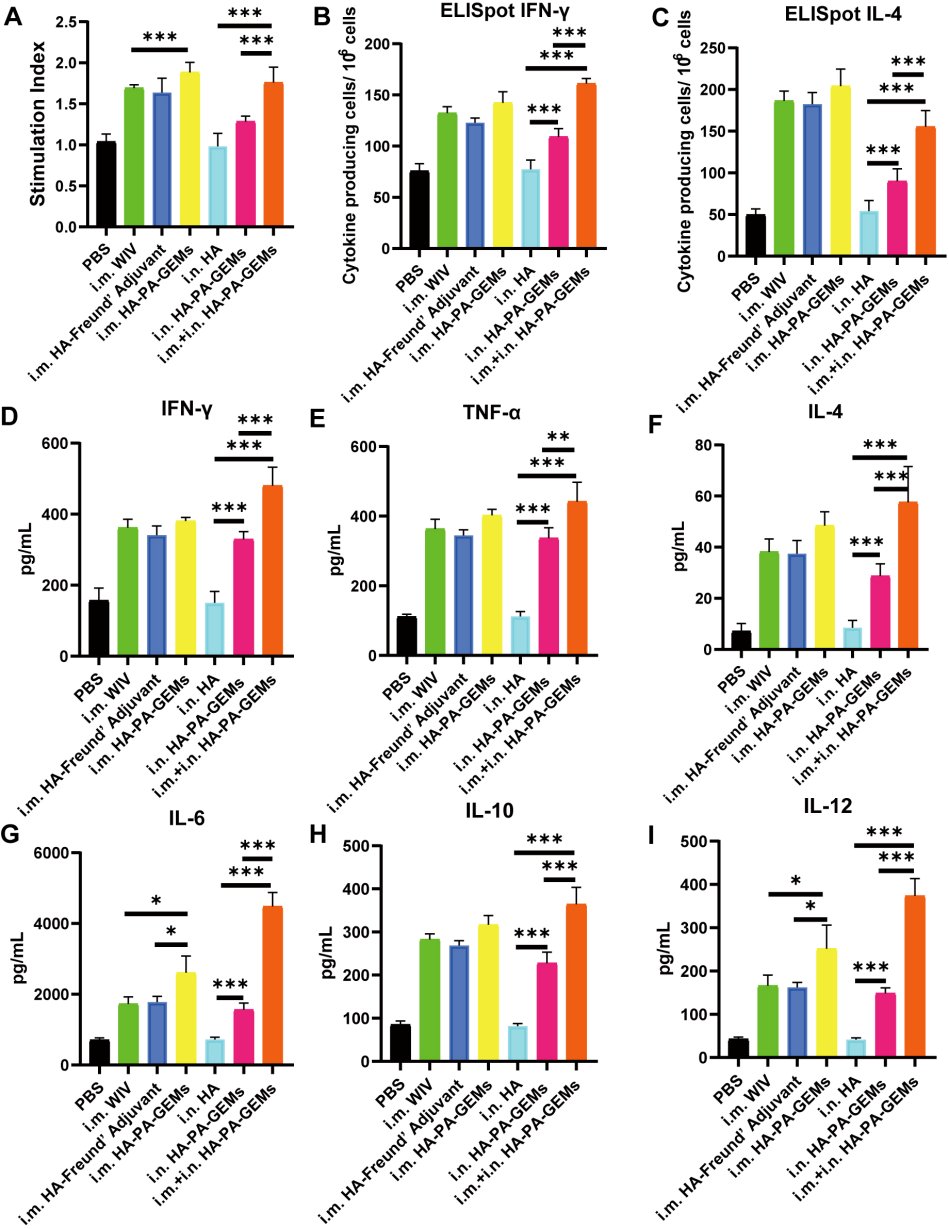
Detection of cellular responses in mice immunized with HA-PA-GEMs. **(A)** Splenocyte proliferation analysis. The stimulation index of the splenocytes was detected using a Cell Counting Kit-8 assay, upon stimulation with purified H1N1 HA protein. The levels of **(B)** INF-γ and **(C)** IL-4 secreted by the splenocytes were quantified using an ELISpot assay. The levels (pg/mL) of the cytokines **(D)** INF-γ, **(E)** TNF-α, **(F)** IL-4, **(G)** IL-6, **(H)** IL-10, and **(I)** IL-12 in the cell-free supernatants harvested from the splenocytes at 48 h after incubation were measured using commercial ELISA. The analysis was performed with a one-way ANOVA with Bonferroni’s *post-hoc* multiple comparison test. Differences were considered significant at *p<0.05, **p<0.01, ***p<0.001. ELISpot assay, enzyme-linked immunosorbent spot assay; HA, hemagglutinin; PA, protein anchor; GEM, gram-positive enhancer matrix; IL, interleukin; ELISA, enzyme-linked immunosorbent assay; INF-γ, interferon gamma; TNF-α, tumor necrosis factor alpha.

The effect of the immunostimulatory agents on the proliferative response of splenocytes was assessed 12 d after the last vaccination. Splenocytes from the inoculated animals multiplied more quickly after H1N1-HA protein stimulation *ex vivo* than those from control mice (*p*<0.01) (**Figure 7A**). Additionally, the i.m. HA-PA-GEM group and i.m.+i.n. HA-PA-GEM group exhibited the highest increase in cell proliferation (*p*<0.001). **Figures 7B, C** shows the identification of the splenocytes from the infected mice that generated IFN-γ and IL-4 specific to the antigen, in order to further evaluate the kind of immune response. ELISpot assays were used to measure IFN-γ and IL-4 secretion by mouse splenocytes (**Figures 7B, C**). These results showed that spot-forming cells, which are indicators of the production of IFN-γ by splenocytes, were significantly higher in mice immunized with i.m.+i.n. HA-PA-GEM than those immunized with i.m. or i.n. HA-PA-GEM (*p*<0.001), thus demonstrating that both the Th1 and Th2 arms of adaptive immunity were activated. Next, we examined the capacity of immune cells extracted from the spleens of immunized mice to release cytokines in response to *ex vivo* stimulation with the H1N1-HA protein, to evaluate the cellular immunological response induced by the HA-PA-GEM experimental vaccines. The levels of IFN-γ, TNF-α, IL-4, IL-6, IL-10, and IL-12 secreted by splenocytes were assayed using commercial ELISA kits. Compared to the animals in the other groups, the mice immunized with i.m.+i.n. HA-PA-GEM released significantly higher quantities of the cytokines IFN-γ, TNF-α, IL-4, IL-6, IL-10, and IL-12 from their splenocytes (*p*<0.001) (**Figures 7D–I**). IFN-γ, TNF-α, IL-6, and IL-12 production was linked to a Th1 profile, whereas IL-4 and IL-10 secretion was linked to a Th2 immune response. These findings showed that HA-PA-GEM vaccination increased the splenocyte production of both type 1 and type 2 cytokines.

### 3.7 Protective efficacy against G4 EA H1N1 challenge

**Figure 8:**
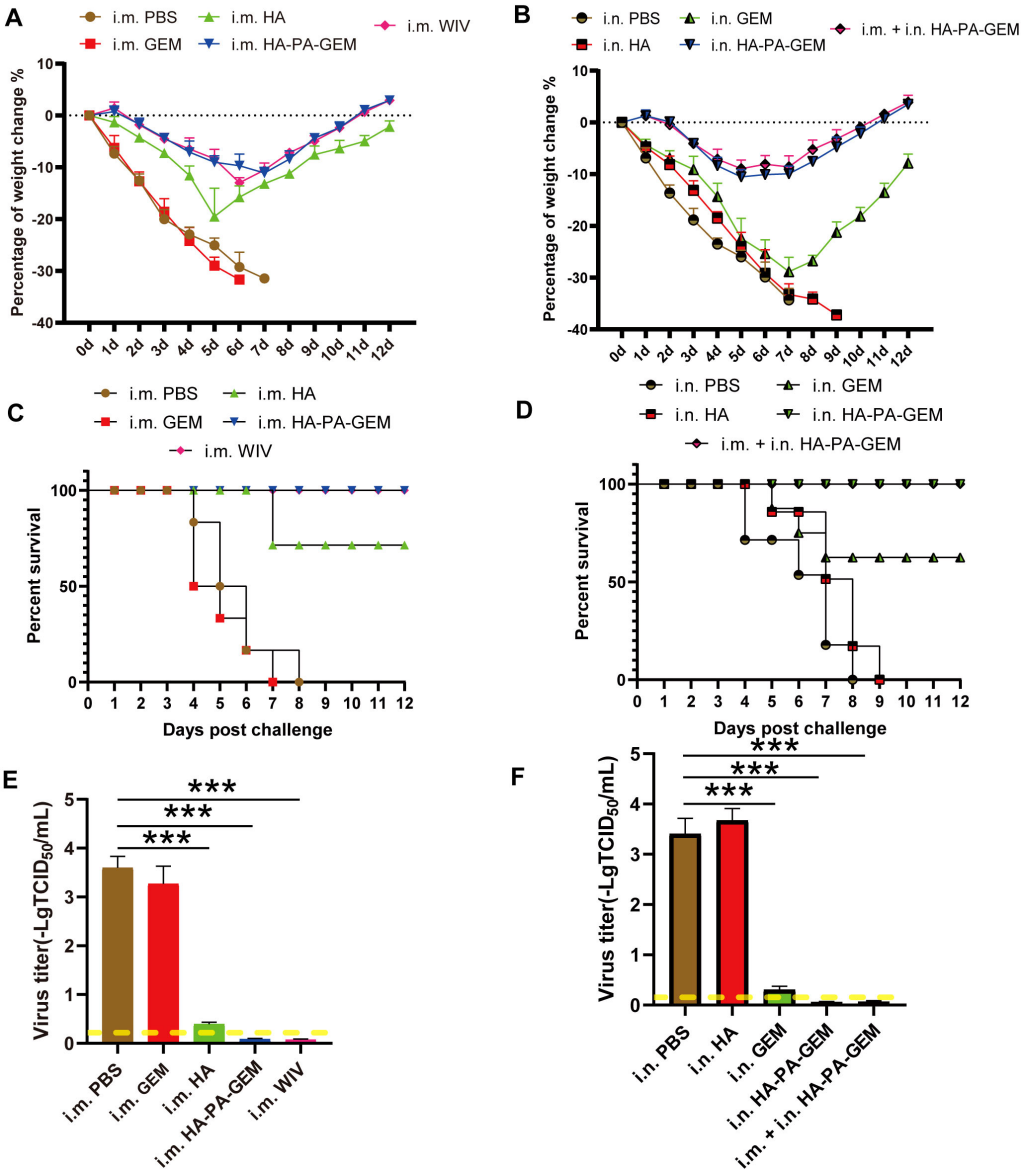
Immunization and challenge studies.Seven days after the last immunization, all the mice in the study group were i.n. challenged with the H1N1 virus. **(A)** Body weight and **(C)** survival of mice i.m. administered PBS, HA, GEM, or HA-PA-GEM were monitored daily for 12 d following the virus challenge; **(B)** Body weight and **(D)** survival of mice administered i.n. PBS, HA, GEM, HA-PA-GEM, or i.m. HA-PA-GEM were monitored daily for 12 d following virus challenge. Virus titers in the lungs of mice **(E)** i.m. injected with PBS, HA, GEM, or HA-PA-GEM and **(F)** i.n. injected with PBS, HA, GEM, HA-PA-GEM, or HA-PA-GEM, at DPC 7. The dotted yellow line shows the limit of detection. The analysis was performed with a one-way ANOVA. Differences were considered significant at ***p<0.001. DPC, days post-challenge; i.n., intranasal; i.m., intramuscular; PBS, phosphate-buffered saline; PA, protein anchor; GEM, gram-positive enhancer matrix; HA, hemagglutinin.

To determine whether the HA-PA-GEM vaccine was protective, the mice (**Supplementary Table 2**) were i.n. infected with G4 EA H1N1 1 week after the third repeated immunization. Following that, the body weights (**Figures 8A, B**) and survival rates (**Figures 8C, D**) of the animals (8 mice per group) were recorded daily for two weeks. These findings demonstrated that immunization of mice with i.m. WIV, i.m. HA, i.m. HA-PA-GEM, i.n. HA-PA-GEM, and i.m.+i.n. HA-PA-GEM prevented weight loss and mortality. A challenge with i.m. GEM and i.n. HA resulted in all mice hitting clinical endpoint within 10 d of immunization. The mock-vaccinated (PBS) group as well as those immunized with i.m. GEM and i.n. HA exhibited lung viral titers of approximately 7×10^3^ TCID_50_/mL on day 7 post-challenge, whereas mice immunization with i.m. WIV, i.m. HA, i.m. HA-PA-GEM, i.n. HA-PA-GEM, and i.m.+i.n. HA-PA-GEM showed no detectable virus in the lungs (**Figures 8E, F**). In addition, on day 7 post-exposure, the challenge virus titer in the lungs of the mice i.n. inoculated with GEM particles was lower than that in the lungs of the mock-vaccinated mice, indicating that the i.n. GEM particles vaccinated mice shed or replicated less in their lungs.

### 3.8 Histopathological analysis of the lungs of mice challenged with G4 EA H1N1

**Figure 9:**
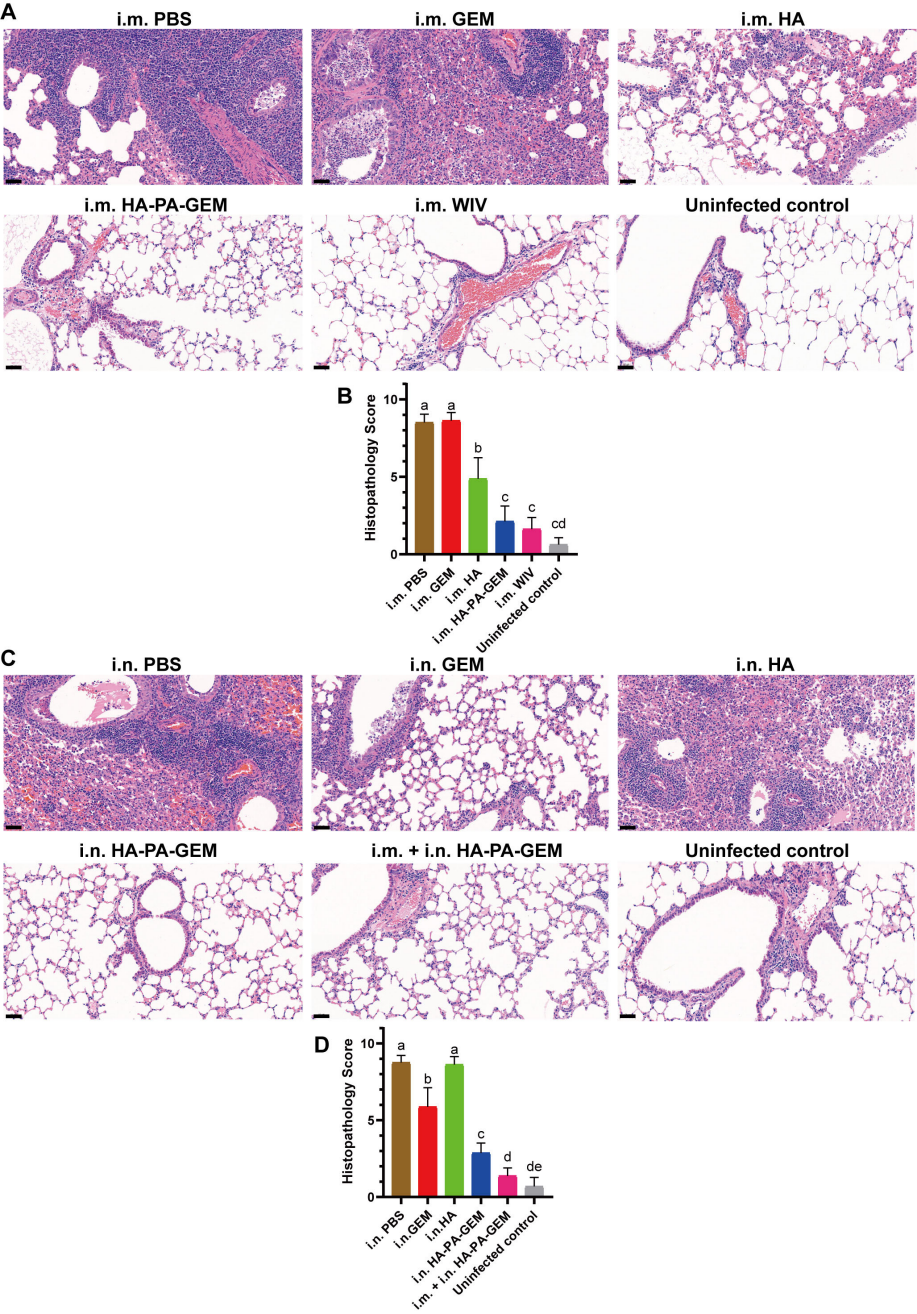
H&E-stained histopathological lesions in the lungs of H1N1-challenged mice. H&E staining for the **(A, B)** i.m. and **(C, D)** i.n administered groups; Scale bar (black): 200 μm. Differences were considered statistically significant at p<0.05. Different letters represent significant differences, and same letters represent no significant differences. H&E, hematoxylin and eosin; i.m., intramuscular; i.n., intranasal; PBS, phosphate-buffered saline; GEM, gram-positive enhancer matrix; HA, hemagglutinin; PA, protein anchor.

We exposed the mice to the G4 EA H1N1 virus after vaccination and conducted a histological investigation on day 7 after the challenge, to determine whether the HA-PA-GEM-elicited antigen-specific mucosal, humoral, and cell-mediated immunity in mice led to pulmonary tissue damage (**Figure 9**). Lung pathology images (**Figure 9A**) revealed that the vaccination groups (i.m. WIV, i.m. HA, and i.m. HA-PA-GEM) had significantly fewer infiltrating cells in the mouse lung tissues, and the alveoli were clearly visible. However, in the control groups, the number of infiltrating cells of the alveolar cavity increased, the alveolar wall thickened, and the majority of the alveolar contours vanished (i.m. PBS and i.m. GEM). I.m. WIV- and HA-PA-GEM-immunized groups exhibited similar histopathological scores (**Figure 9B**). On day 7 post-challenge, the lungs stained with hematoxylin and eosin revealed noticeably more inflammation in the PBS-inoculated group and in those that received i.n. HA vaccination than in those that received i.n. HA-PA-GEM vaccination. Moreover, i.n. HA-PA-GEM and i.m.+i.n. HA-PA-GEM immunizations caused the least amount of inflammation at seven days post-challenge, as compared to that observed in the PBS- and HA-treated groups (**Figure 9C**). The i.m.+i.n. HA-PA-GEM group showed significantly (*p*<0.01) lower histopathological scores at 7 d post-challenge than the other groups (**Figure 9D**). Thus, the HA-PA-GEM vaccination may successfully prevent the H1N1 influenza virus from harming mouse lung tissues.

### 3.9 Protection against lethal PR8 virus challenge

In PR8 H1N1 challenge studies, the i.m. WIV- and i.m. HA-PA-GEM-immunized animals exhibited weight loss of >15%, while the i.n. and i.m.+i.n. HA-PA-GEM-immunized animals exhibited a reduced weight loss of <10%, as shown in **Supplementary Figures 7A–B**. The results demonstrate that the animals immunized via the intramuscular (i.m.) routes with the HA-PA-GEM vaccine exhibited moderate cross-protective efficacy against the PR8 H1N1 strain. However, the animals immunized via the intranasal (i.n.) routes with the HA-PA-GEM vaccine demonstrated superior cross-protective efficacy against the PR8 H1N1 strain. This may be attributed to the role of mucosal immunity. This also provides a promising indication that GEM, when used as an adjuvant through intranasal immunization, can achieve a favorable immunoprotection rate. When challenged with the PR8 virus, the mice i.m. immunized with WIV and HA-PA-GEM displayed survival rates of 46%–52% (**Supplementary Figure 7C**). We found fewer viruses in the lung samples from the i.m. WIV- and HA-PA-GEM-immunized animals than those from the mock-vaccinated mice (**Supplementary Figure 7E**). As supported by the 100% survival rate, the i.n. HA-PA-GEM- and i.m.+i.n. HA-PA-GEM-immunized groups exhibited significantly better protection against deadly PR8 viral infections (**Supplementary Figure 7D**). In addition, the mice in the i.n. HA-PA-GEM- and i.m.+i.n. HA-PA-GEM-immunized groups showed significantly lower mean viral lung titers than those observed in the mock-vaccinated animals (**Supplementary Figure 7F**). Therefore, these results showed that both the i.n. and i.m.+i.n. HA-PA-GEM-immunized groups conferred cross-protection against PR8 IAVs.

## 4 Discussion

Pigs are crucial hosts for studying and managing mammalian influenza viruses. It is common for humans and pigs to come in contact with each other, leading to interspecies transmission and reassortment, which can in turn result in the development of new influenza strain (30–32). There is a need to regularly update the vaccine formulation for swine IAV immunization to act as an effective control measure to lower the disease burden and pandemic influenza risk (30–32). Utilizing adjuvants is a crucial tactic for improving immunogenicity and antigen sparing (18, 33). In this study show that GEM particles can be used as adjuvants for i.n. and i.m. delivery of influenza subunit vaccines. The inclusion of GEM particles in influenza subunit vaccination markedly improved the systemic and mucosal immune responses. Additionally, GEM particles improve immune response quality by ensuring a balanced Th1/Th2-type response, instead of a Th2-dominated response (18). These results are in keeping with previous observational studies, which the intravenously delivered subunit vaccines were more immunogenic when delivered with GEM particles (13, 34–40). This study is the first to demonstrate that GEM particles in swine influenza subunit vaccines significantly enhance both systemic and mucosal immune responses. These results will be widely beneficial to both the biomedical and veterinary fields.

Previous studies have reported that the following benefits for GEM surface display systems: (1) Easy purification of foreign proteins (38); (2) Generally, *L. lactis* is regarded as a safe probiotic. After treatment, GEM particles pose no safety risks as a carrier, because they do not contain proteins or nucleic acids (38, 41, 42); (3) GEM particles can effectively boost the immune system’s reaction (19, 38). Consequently, this method has been used in numerous vaccine studies (38, 43, 44). Macrophages are crucial components of the innate immune system that help the host defend itself against infections and launch immunological responses (24, 45). Macrophages are the antigen-presenting cells that are responsible for triggering adaptive immunity by releasing inflammatory cytokines (IL-1, IL-6, *etc.*) and chemokines (TNF-α, IFN-γ, *etc.*) (24, 46, 47). This study found that GEM particles led to a significant increase in nitrite production in RAW 264.7 cells, indicating their activation. In addition, after stimulation with GEM, the gene expression data showed higher levels of iNOSl and cytokines, similar to those observed upon treatment with the Pam3CK4 control. Overall, this study strengthens the idea that GEM particles are potent stimulators of innate immunity. These findings align with numerous prior studies on Feline herpesvirus 1 (35), C. perfringens (CPMEA) (48), and Canine distemper virus (CDV) (34). Peptidoglycan, a key component of BLPs, binds to TLR2, which forms a heterodimer with TLR1 or TLR6 to activate innate immune cells (49). Ramirez et al. demonstrated that GEMs interact solely with HEK293 cells expressing human TLR2, triggering NF-*κ*B activation (50). Research is being conducted on GEMs-activated TLR receptors *in vivo*, including a study with GEMs mixed with a split influenza vaccine. Nasally immunized TLR2KO mice exhibited reduced serum IgG, lower sIgA in nasal and lung lavages, and fewer IFN-γ producing T-cells and B-cells in local dLN and spleen compared to wild-type controls (20, 49). In this study we assess the adjuvant properties of GEM particles, mice were i.n. administered a single dose of native GEM particles in PBS. The findings showing that these mice had large, organized lymphoid follicles in both lungs and nasal cavities. These tissues, also called bronchus- and nasal-associated lymphoid tissues, contain dendritic cells, T cells, and a significant number of B cells.

Commercially available inactivated SIV vaccines offer only a modest level of protection against heterologous viruses, although they provide sterile immunity against homologous viruses (32, 51). Therefore, there is an urgent need to develop alternative inactivated vaccine platforms. The adjuvant properties of GEM (gram-positive enhancer matrix) particles have garnered significant attention in recent years due to their potential to enhance immune responses in various vaccination strategies. GEM particles serve not only as carriers for antigens but also as immunostimulants that can modulate the immune system effectively. For instance, GEM particles have been utilized to display antigens, such as the E2 glycoprotein of the classical swine fever virus, enhancing the immunogenicity of the vaccine formulations. The E2-Spy-PA-GEM complex has shown to induce high levels of antibodies in immunized mice, indicating that GEM particles can significantly improve the immune response to specific antigens (52). Further, Numerous studies have shown that the use of GEMs for i.n. vaccination has many benefits over the use of inactivated vaccines. This platform is a strong alternative to the traditional inactivated vaccines used in swine, because of its quick turnaround time, stimulation of an immune response similar to that caused by a natural route of infection, lack of need for an additional adjuvant, and effectiveness with a single dose (18, 38). In this study, we found that the presence of GEM particles increased HI titers, improved the Th1/Th2-type immune response, decreased lung viral titers, and completely cleared the virus by day 7 post challenge. Moreover, we demonstrated that i.n. immunization of mice with influenza HA subunit vaccine+GEM complexes enhanced mucosal and humoral immune responses, as compared to that observed upon immunization of mice with subunit vaccine alone. The increased viral clearance in mice given adjuvanted vaccination may be due to the generation of higher IgG2a titers and a comparatively high IFN-γ/IL-4 ratio. In contrast, the i.m.+i.n. HA-PA-GEM influenza vaccine induced a skewed response toward the Th1 type. Leenhouts et al. extensively studied GEM’s impact on boosting influenza vaccine immunity. They found that adding GEMs to an intranasally administered H1N1 vaccine significantly increased serum IgG, HI levels, and sIgA titers in mice, providing complete protection against both homologous and heterologous influenza infections and reducing lung viral titers (17). Oral immunization with a monovalent subunit vaccine of strain A/Hiroshima (H3N2) mixed with GEMs induced stronger systemic and local antibody responses and a more balanced Th1/Th2 response than inactivated influenza vaccines with alum. Similarly, combining GEMs with seasonal HA (A/Wisconsin/67/2005 [H3N2]) significantly enhanced systemic immune responses and achieved a more balanced Th1/Th2 response compared to HA alone administered intramuscularly (19). Our results corroborate the findings of a great deal of the previous work in nasal immunization with a subunit vaccine combined with GEMs reduced IL-4-producing cells and significantly increased IFN-γ-producing cells, shifting the immune response to a Th1-type. Moreover, GEMs-based vaccines showed promising safety and immunogenicity in the FluGEM phase I trial (42).

Mucosal immunization, by emulating the natural invasion pathways of pathogens, stimulates mucosal immunity through the production of secretory immunoglobulin A (sIgA) and elicits a systemic immune response characterized by immunoglobulin G (IgG) production (53). Furthermore, this needle-free approach to immunization minimizes immune-related side effects and is well-suited for large-scale vaccination initiatives and repeated immunization protocols (54). As most viruses enter and begin their reproduction at mucosal surfaces, mucosal vaccination is highly desired to increase protection against infectious illnesses. The size of GEMs is similar to that of live *L. lactis*, around 1 μm, making them suitable for uptake by M cells in the nasal-associated lymphoid tissue (NALT) (55). Researchers have confirmed that after i.m. vaccination, most mice have SIgA levels in lung lavage that are below the limit of detection (56–58). Using different immunization route, we explored whether HA-PA-GEM vaccination induces a mucosal SIgA response in this study. In the present study, it was found that mice immunized with i.n. or i.m.+i.n. HA-PA-GEM showed high SIgA titers in lung lavages after i.n. administration. In accordance with the present results, previous studies have demonstrated that that i.n. vaccination with GEM vaccines enhances SIgA levels in BALB/c mice, as compared to parenteral vaccination (19, 20, 52). This intriguing result might be explained by the fact that HA-PA-GEM vaccination could drive the entire mucosal immune system. An *in vitro* assay showed that GEMs activate human nasal epithelial cells, increasing IL-6 and IL-8 levels (59–61). To explore M cell interaction with GEMs, mice were intranasally given fluorescent GEMs. After 15 minutes, staining revealed that M cells efficiently absorbed GEMs (59–61). Additionally, some lamina propria DCs extend dendrites through the epithelium to directly capture antigens (61). These findings will help others to better understand the GEMs vaccination of the local mucosa activates the whole mucosal system.

In this study, cellular and humoral immunological responses to influenza virus infection were induced by the HA-PA-GEM vaccines, thus showing that they effectively stimulate the immune system. In every instance, the vaccinated mice produced hemagglutinating antibodies against the relevant vaccination antigen. The EA H1N1 SIV HA is generated from influenza viruses of avian origin that have little cross-reactivity with seasonal or classic swine H1N1 viruses (62, 63). Antigenic alterations in HA may result from mutations, particularly in the HA1 region. Consequently, in the current study, G4 EA H1N1 and PR8 viruses were used as challenge viruses to assess the efficacy of HA-PA-GEM immunization against homologous and heterologous viral infections in mice. In addition to providing total protection against homologous viral infections, inoculation with i.n. or i.m.+i.n. HA-PA-GEM resulted in strong cross-protection against heterologous H1N1 viral infections. It should be noted that the experimental animals used in this experiment were mice, rather than pigs. Consequently, further validation of these data is required on a porcine model, which is a key objective of our future research program. GEMs are an efficient, cost-effective, and safe tool, particularly useful in subunit vaccine development due to their simple preparation and antigen display system. However, enhancing GEMs vaccine efficacy is still challenging. Future advancements in GEMs display systems will likely rely on improving vaccine performance.

## 5 Conclusion

HA-PA-GEMs triggered specific immune responses in BALB/c mice. Intramuscular administration led to strong serum IgG responses and a higher IgG2a/IgG1 ratio but did not enhance systemic or mucosal SIgA responses. In contrast, intranasal immunization with HA-PA-GEMs, unlike HA alone, effectively induced systemic and mucosal SIgA responses and significantly boosted IFN-γ responses, promoting Th1-type immunity. As a result, HA-PA-GEMs stand out as an innovative and promising vaccine candidate for protecting veterinary animals from influenza viruses. This vaccine offers several advantages over alternative methods, including safety, appropriateness of mucosal administration (which makes it practical for widespread use), robust immunogenicity, and broad protective capacity. The findings of this study call for further investigation of this hypothesis in swine.

## Data Availability

The original contributions presented in the study are included in the article/**Supplementary Material**. Further inquiries can be directed to the corresponding author.

## Supplementary material

Supplementary Figure 1 Experimental immunization and challenge diagram.

Supplementary Figure 2 Physical and biological characterization of GEM particles. **(A)** MG1363 and GEM staining gram-positive (magnification: 1000×). **(B)** Transmission electron microscopy analysis. Untreated MG1363 cells and the GEM particles obtained from MG1363 cells. **(C)** Scanning electron microscopy analysis. Particle size of **(D)** MG1363 cells and the GEM particles obtained from MG1363 cells.

Supplementary Figure 3 H&E staining of cross-sections revealed the histological characteristics of the murine nasal, lung parenchyma, and mediastinal adipose tissues. Mice were intranasally administered with PBS alone or native GEMs in PBS. Five days later, the heads and thoracic tissues obtained from the animals were processed for H&E staining. **(A, A1, D, D1)** murine nasal tissues; **(B, B1, E, E1)** lung parenchyma tissues; and **(C, C1, F, and F1)** mediastinal tissues. Scale bar: red=1 mm, green=200 μm. H&E, hematoxylin and eosin.

Supplementary Figure 4 The cytotoxic effects of various doses of gram-positive enhancer matrix particles administered through the intranasal route. **(A)** 2000 U (1:0); **(B)** 200 U (1:10); **(C)** 20 U (1:100); **(D)** 2 U (1:1000); **(E)** 1 U (1:2000); **(F)** Quantitative analysis of the hematoxylin and eosin staining. Scale bar: 1 mm. U, unit.

Supplementary Figure 5 Fusion proteins on the surface of GEM particles were detected using IFA. **(A)** Immunofluorescence was detected as green fluorescence using anti-6×His-tag monoclonal antibody and FITC-conjugated goat anti-mouse antibody, while **(B)** red fluorescence was detected in samples seropositive from swine H1N1, upon staining with DyLight 594-conjugated goat anti-swine IgG.

Supplementary Figure 6 HA-specific IgG subclass binding antibody responses. **(A)** IgG2a/IgG1 ratio; **(B)** IgG2b/IgG1 ratio.

Supplementary Figure 7 Weight changes and percent survival in mice after challenge with live virus (A/PR/8/34). Groups of immunized and unimmunized mice intranasally challenged with live virus (A/PR/8/34), at 1 week after the third immunization. Average body weight changes **(A, B)** and survival rates **(C, D)** were monitored for 12 d. The lungs of each mouse group were collected for **(E, F)** viral titer detection, at 7 days post-challenge in MDCK cells, and the results were calculated as log10. The dotted yellow line shows the limit of detection. The analysis was performed with a one-way ANOVA. Differences were considered significant at *p<0.05, **p<0.01, ***p<0.001. PBS, phosphate-buffered saline; WIV, whole-inactivated virus; HA, hemagglutinin; GEM, gram-positive enhancer matrix.

Supplementary Table 1 Primers sequences and real-time PCR amplification parameters.

Supplementary Table 2 Animal study design.
