# Supplementary figures and images for: A gram-positive enhancer matrix particles vaccine displaying swine influenza virus hemagglutinin protects mice against lethal H1N1 viral challenge
Source: Front Immunol. 2025 Jan 6;15:1432989. doi: 10.3389/fimmu.2024.1432989 (PMC11743504; doi:10.3389/fimmu.2024.1432989)

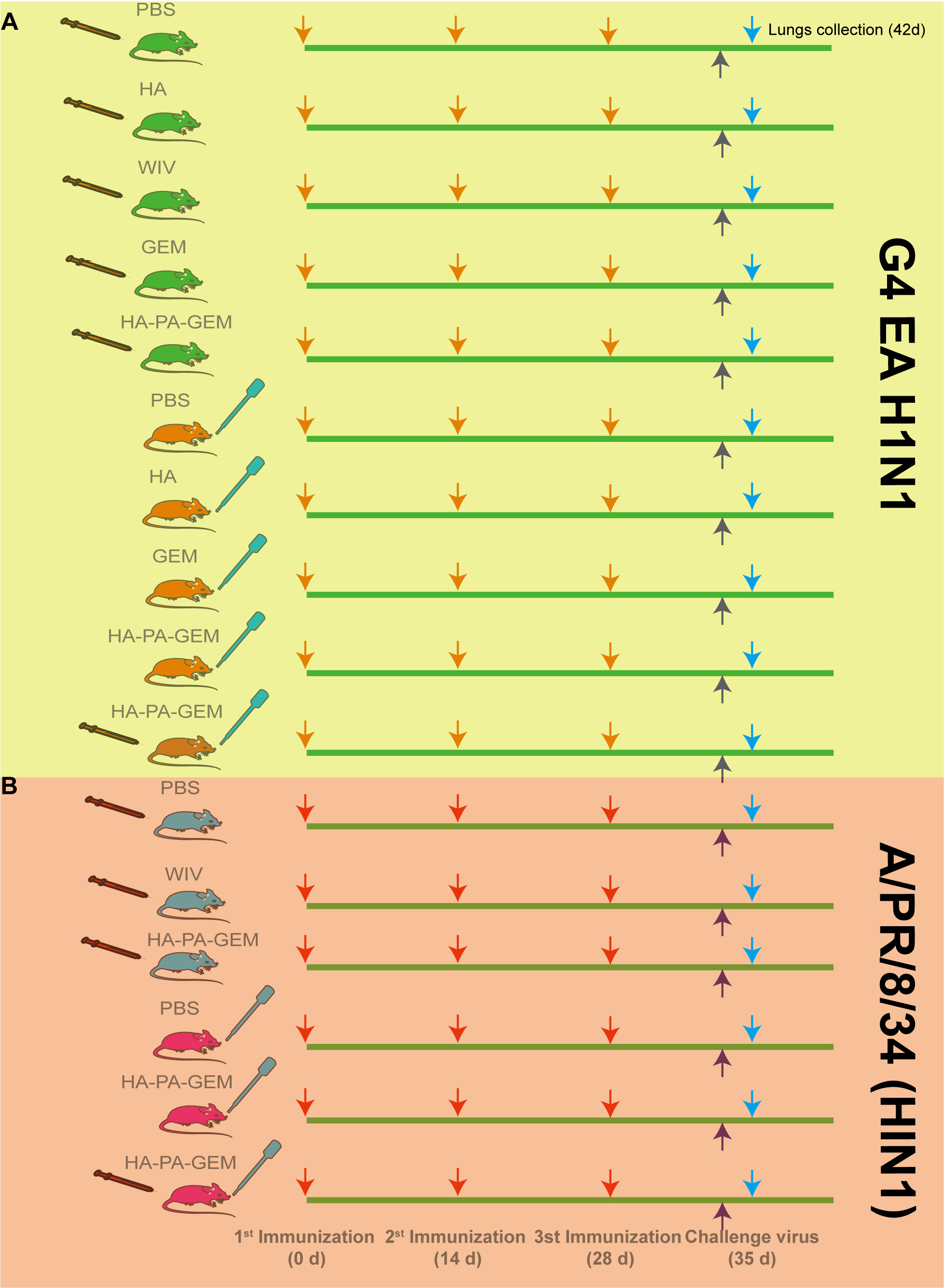

Supplement: Supplementary Figure 1 — Experimental immunization and challenge diagram. [file Image1.jpeg]

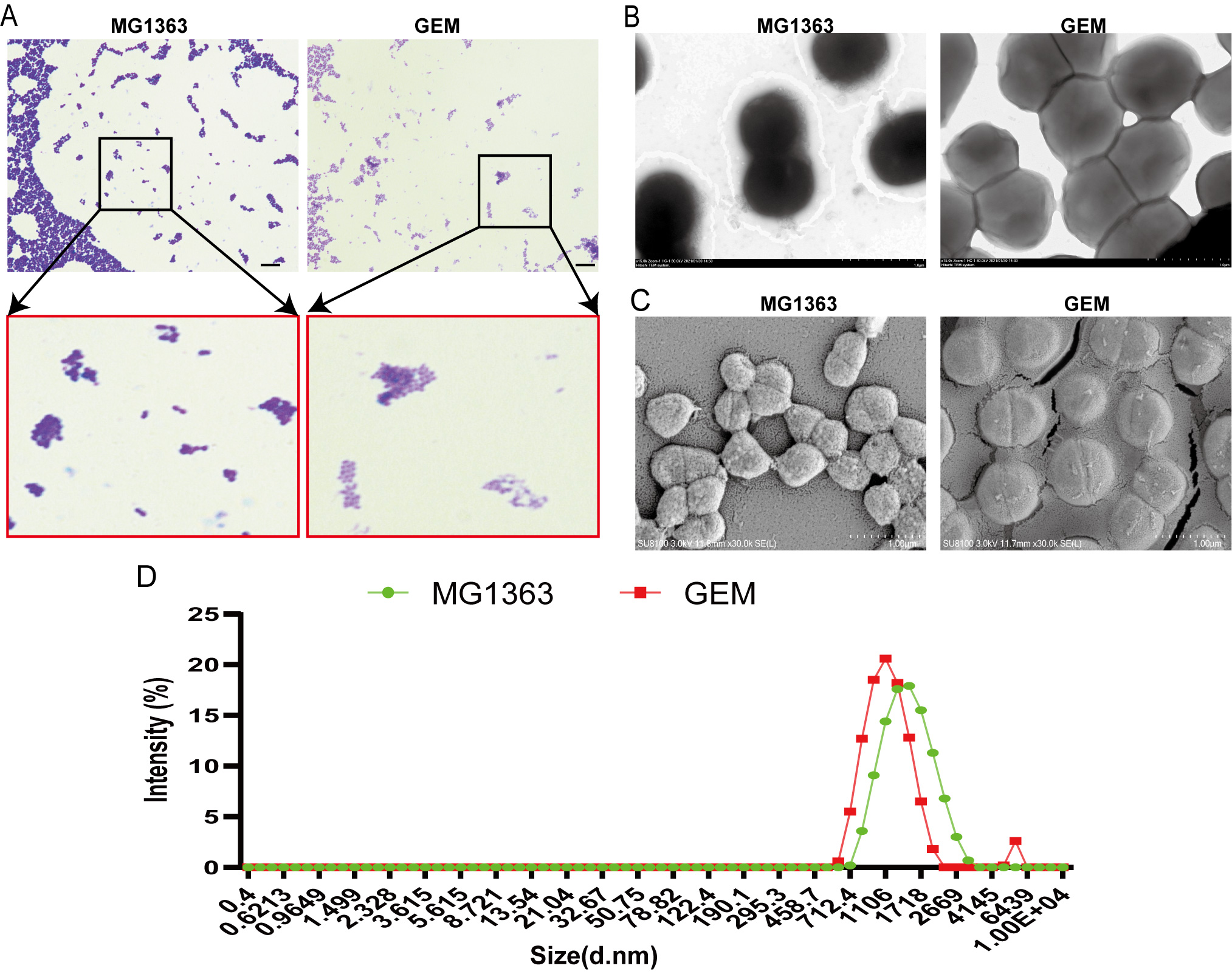

Supplement: Supplementary Figure 2 — Physical and biological characterization of GEM particles. (A) MG1363 and GEM staining gram-positive (magnification: 1000×). (B) Transmission electron microscopy analysis. Untreated MG1363 cells and the GEM particles obtained from MG1363 cells. (C) Scanning electron microscopy analysis. Particle size of (D) MG1363 cells and the GEM particles obtained from MG1363 cells. [file Image2.jpeg]

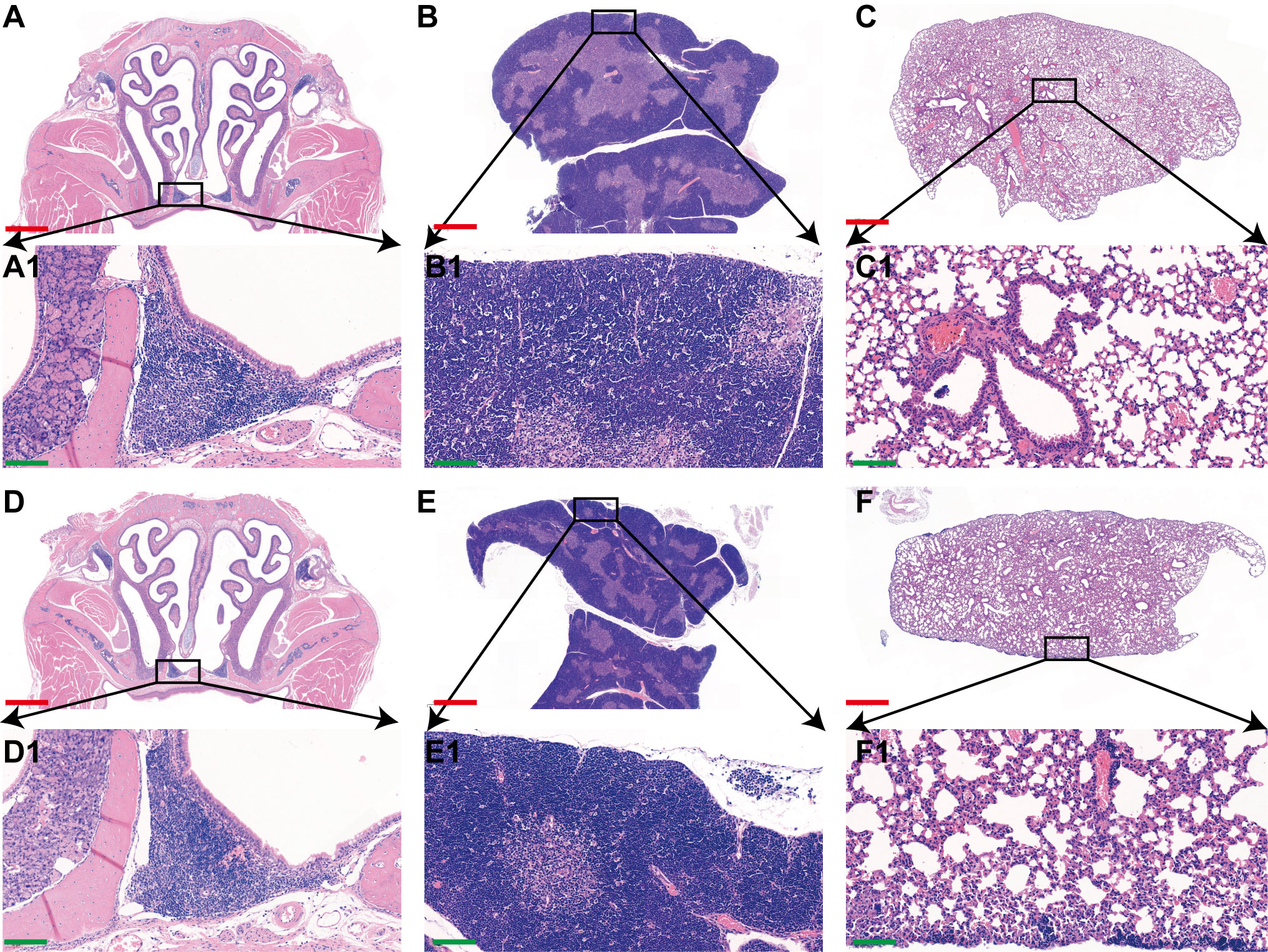

Supplement: Supplementary Figure 3 — H&E staining of cross-sections revealed the histological characteristics of the murine nasal, lung parenchyma, and mediastinal adipose tissues. Mice were intranasally administered with PBS alone or native GEMs in PBS. Five days later, the heads and thoracic tissues obtained from the animals were processed for H&E staining. (A, A1, D, D1) murine nasal tissues; (B, B1, E, E1) lung parenchyma tissues; and (C, C1, F, and F1) mediastinal tissues. Scale bar: red=1 mm, green=200 μm. H&E, hematoxylin and eosin. [file Image3.jpeg]

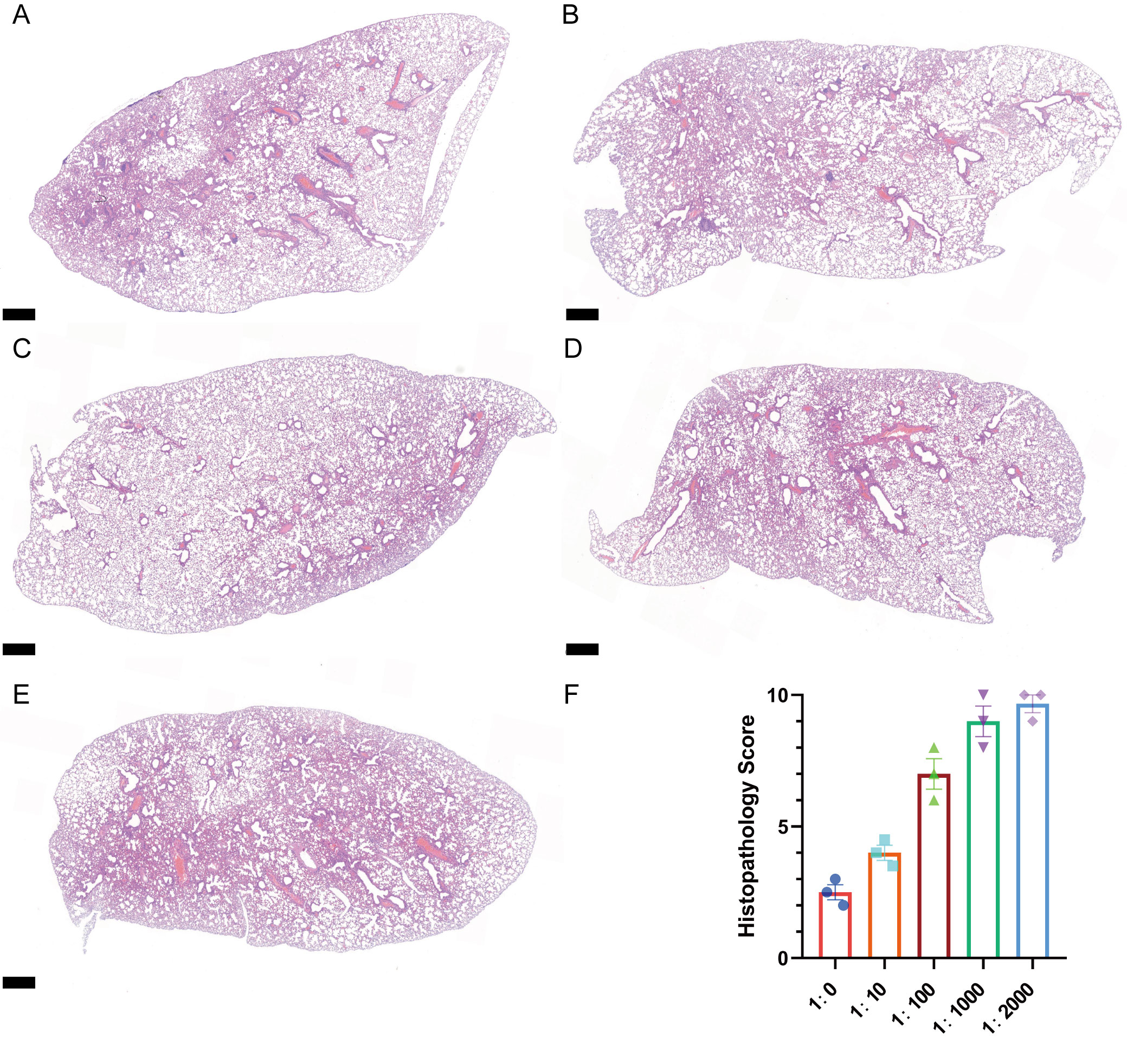

Supplement: Supplementary Figure 4 — The cytotoxic effects of various doses of gram-positive enhancer matrix particles administered through the intranasal route. (A) 2000 U (1:0); (B) 200 U (1:10); (C) 20 U (1:100); (D) 2 U (1:1000); (E) 1 U (1:2000); (F) Quantitative analysis of the hematoxylin and eosin staining. Scale bar: 1 mm. U, unit. [file Image4.jpeg]

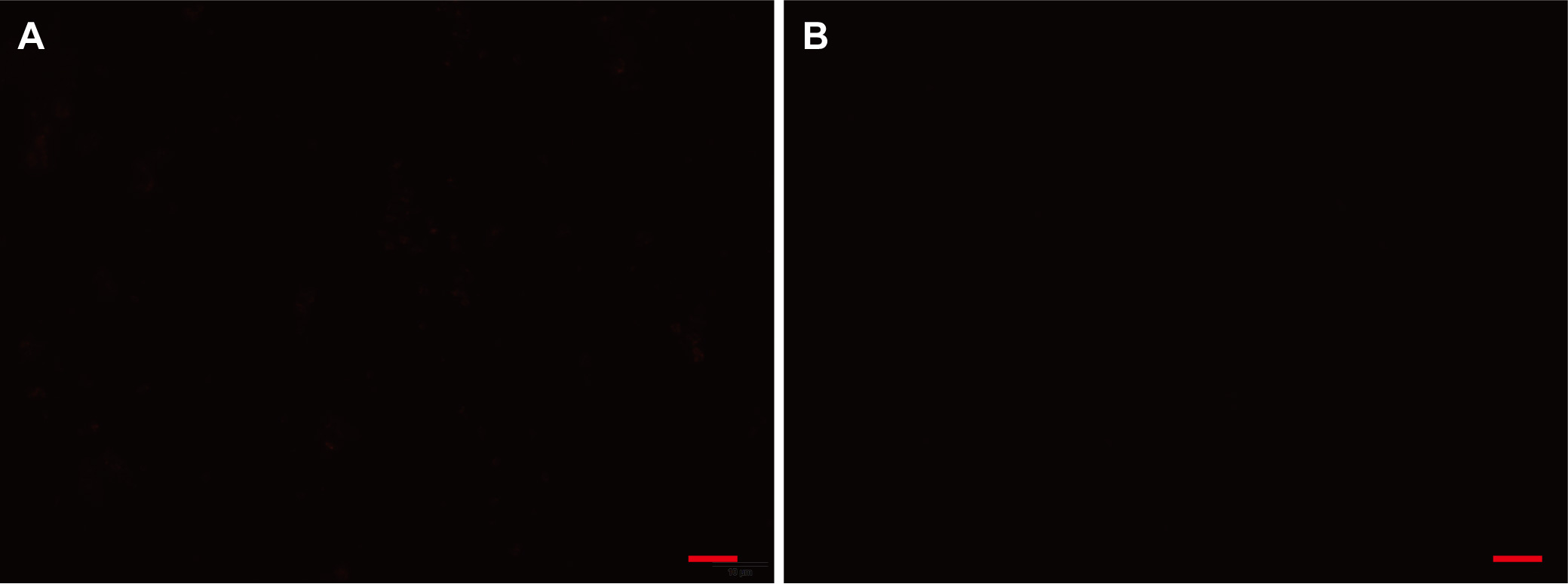

Supplement: Supplementary Figure 5 — Fusion proteins on the surface of GEM particles were detected using IFA. (A) Immunofluorescence was detected as green fluorescence using anti-6×His-tag monoclonal antibody and FITC-conjugated goat anti-mouse antibody, while (B) red fluorescence was detected in samples seropositive from swine H1N1, upon staining with DyLight 594-conjugated goat anti-swine IgG. [file Image5.jpeg]

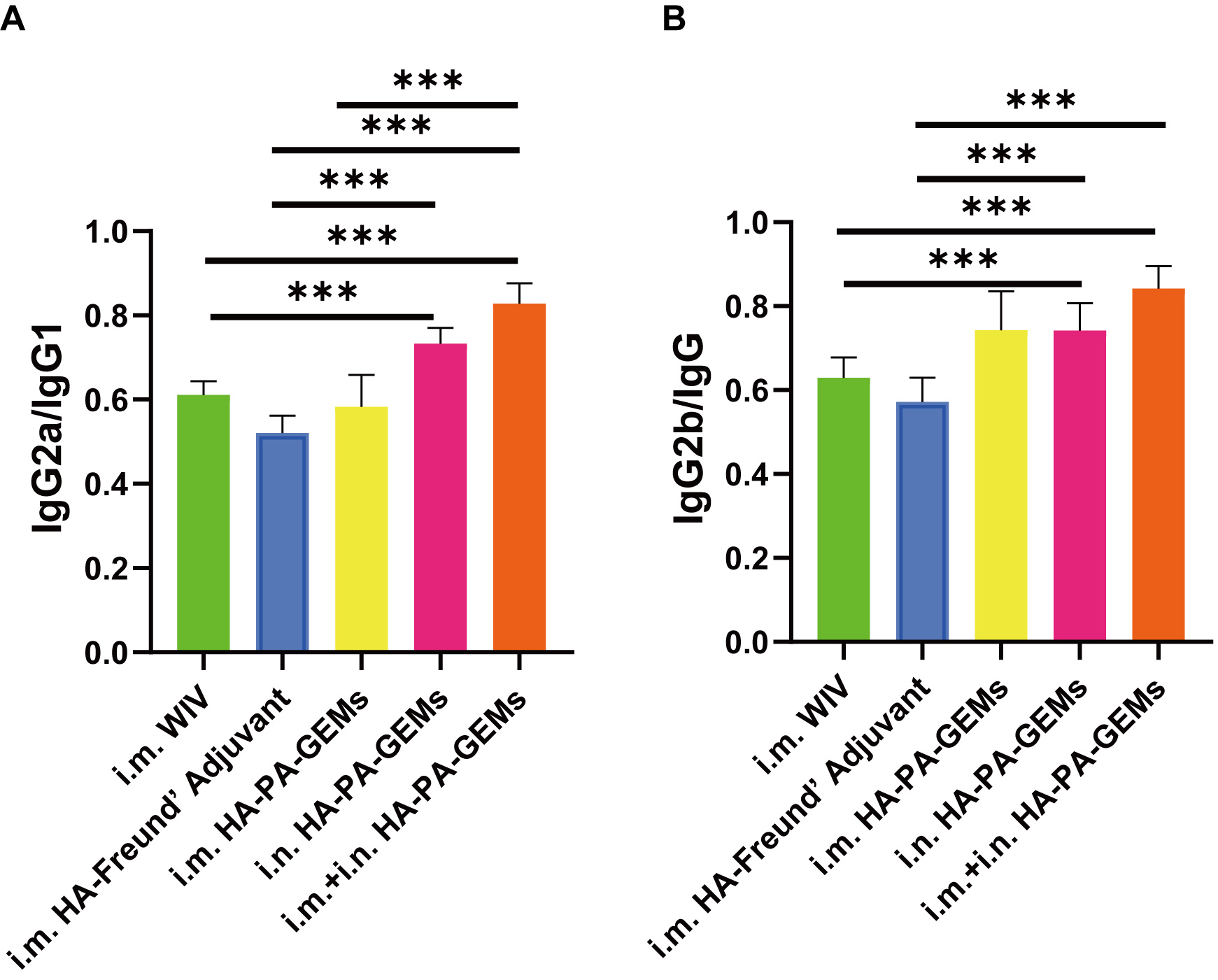

Supplement: Supplementary Figure 6 — HA-specific IgG subclass binding antibody responses. (A) IgG2a/IgG1 ratio; (B) IgG2b/IgG1 ratio. [file Image6.jpeg]

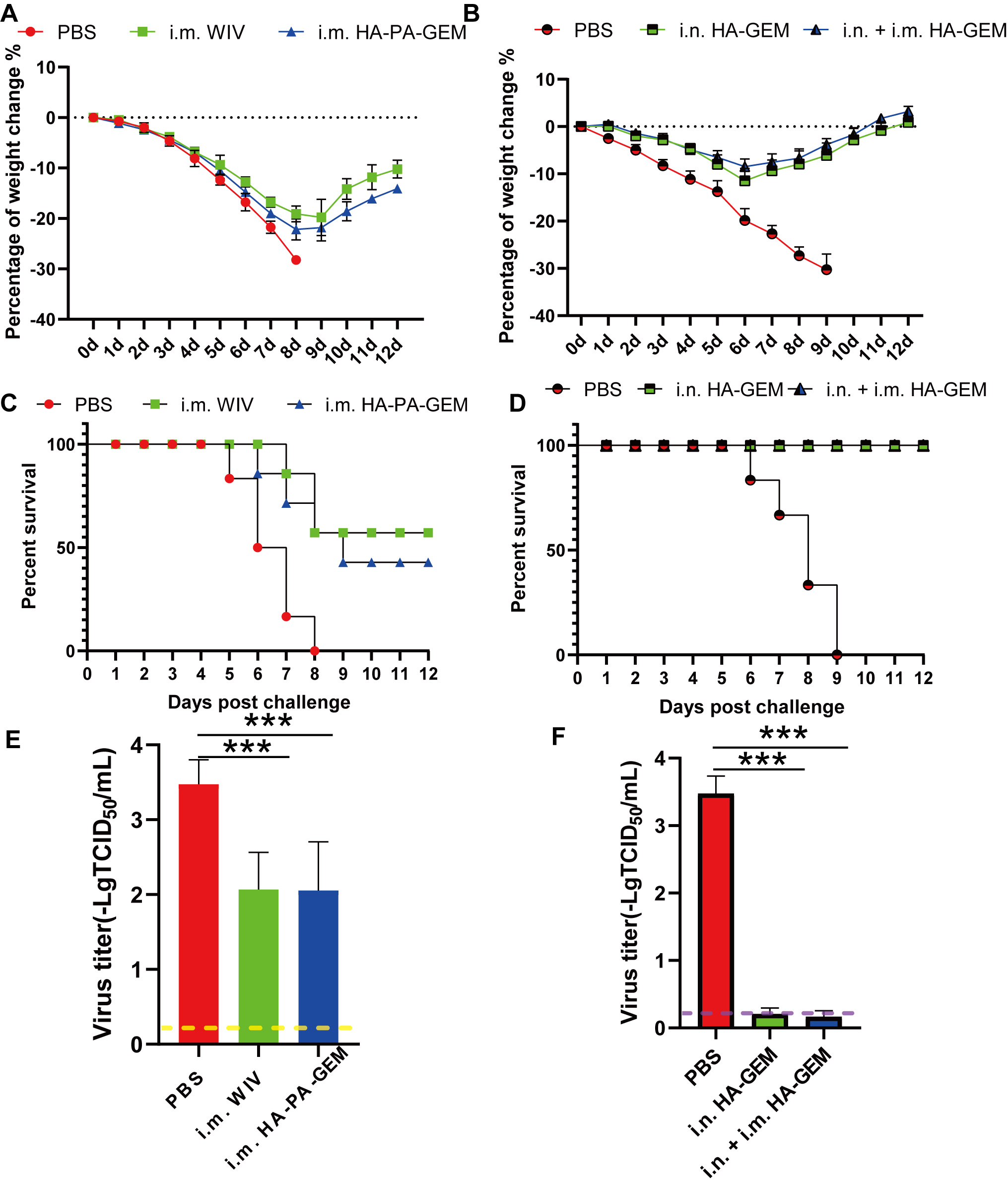

Supplement: Supplementary Figure 7 — Weight changes and percent survival in mice after challenge with live virus (A/PR/8/34). Groups of immunized and unimmunized mice intranasally challenged with live virus (A/PR/8/34), at 1 week after the third immunization. Average body weight changes (A, B) and survival rates (C, D) were monitored for 12 d. The lungs of each mouse group were collected for (E, F) viral titer detection, at 7 days post-challenge in MDCK cells, and the results were calculated as log10. The dotted yellow line shows the limit of detection. The analysis was performed with a one-way ANOVA. Differences were considered significant at *p<0.05, **p<0.01, ***p<0.001. PBS, phosphate-buffered saline; WIV, whole-inactivated virus; HA, hemagglutinin; GEM, gram-positive enhancer matrix. [file Image7.jpeg]
