# Supplementary material for: A gram-positive enhancer matrix particles vaccine displaying swine influenza virus hemagglutinin protects mice against lethal H1N1 viral challenge
Source: Front Immunol. 2025 Jan 6;15:1432989. doi: 10.3389/fimmu.2024.1432989 (PMC11743504; doi:10.3389/fimmu.2024.1432989)
Supplement: Supplementary Table 1 — Primers sequences and real-time PCR amplification parameters. [file Table1.doc]

**Supplementary Table 1 Primers sequences and real-time PCR amplification parameters.**

| **Genes** | **Accession No.** | **Primer (5'-3')** | **Product size/bp** | **Annealing temperature/℃** |
| --- | --- | --- | --- | --- |
| IL-1b | NM_008361.4 | TGCCACCTTTTGACAGTGATG  CAAAGGTTTGGAAGCAGCCC | 84 | 60.0 |
| IL-6 | NM_031168.2 | AGTTCCTCTCTGCAAGAGACTTC  TTTCCACGATTTCCCAGAGAAC | 189 | 60.0 |
| IFN-g | NM_008337.4 | AGGAACTGGCAAAAGGATGGT  CTGGTGGACCACTCGGATG | 258 | 60.0 |
| TNF-a | NM_013693.3 | GGACAGTGACCTGGACTGTG  GAGGCAACCTGACCACTCTC | 127 | 60.0 |
| iNOSl | M87039.1 | AGAGCCACAGTCCTCTTTGC  ACCACCAGCAGTAGTTGCTC | 155 | 60.0 |
