# Supplementary material for: A gram-positive enhancer matrix particles vaccine displaying swine influenza virus hemagglutinin protects mice against lethal H1N1 viral challenge
Source: Front Immunol. 2025 Jan 6;15:1432989. doi: 10.3389/fimmu.2024.1432989 (PMC11743504; doi:10.3389/fimmu.2024.1432989)
Supplement: Supplementary Table 2 — Animal study design. [file Table2.doc]

Supplementary Table 2. Animal study design

| **Study** | **Vaccination modalities** | **Group** | **Vaccine** | **Challenge virus** | **No. of mice** |
| --- | --- | --- | --- | --- | --- |
|  |  |  |  |  |  |
| 1 | Intramuscularly | PBS | No vaccine | G4 EA H1N1 | 8 |
|  | Intramuscularly | PBS | No vaccine | saline | 8 |
|  | Intramuscularly | HA | HA protein | G4 EA H1N1 | 8 |
|  | Intramuscularly | WIV | G4 EA H1N1 | G4 EA H1N1 | 8 |
|  | Intramuscularly | GEM | No vaccine | G4 EA H1N1 | 8 |
|  | Intramuscularly | HA-PA-GEM | HA-PA-GEM | G4 EA H1N1 | 8 |
|  | Intranasally | PBS | No vaccine | G4 EA H1N1 | 8 |
|  | Intranasally | PBS | No vaccine | saline | 8 |
|  | Intranasally | HA | HA protein | G4 EA H1N1 | 8 |
|  | Intranasally | GEM | No vaccine | G4 EA H1N1 | 8 |
|  | Intranasally | HA-PA-GEM | HA-PA-GEM | G4 EA H1N1 | 8 |
|  | Intramuscularly + Intranasally | HA-PA-GEM | HA-PA-GEM | G4 EA H1N1 | 8 |
| 2 | Intramuscularly | PBS | No vaccine | A/PR/8/34 (HIN1) | 8 |
|  | Intramuscularly | PBS | No vaccine | saline | 8 |
|  | Intramuscularly | WIV | G4 EA H1N1 | A/PR/8/34 (HIN1) | 8 |
|  | Intramuscularly | HA-PA-GEM | HA-PA-GEM | A/PR/8/34 (HIN1) | 8 |
|  | Intranasally | PBS | No vaccine | A/PR/8/34 (HIN1) | 8 |
|  | Intranasally | PBS | No vaccine | saline | 8 |
|  | Intranasally | HA-PA-GEM | HA-PA-GEM | A/PR/8/34 (HIN1) | 8 |
|  | Intramuscularly + Intranasally | HA-PA-GEM | HA-PA-GEM | A/PR/8/34 (HIN1) | 8 |
